# Adaptive low-light image enhancement using Interval-Valued Intuitionistic Fuzzy Set optimized by Reptile Search Algorithm

**DOI:** 10.3389/frai.2025.1721291

**Published:** 2026-01-12

**Authors:** Haripriya Yogambaram, M. Sivabalakrishnan, S. Balaji

**Affiliations:** 1 Department of Mathematics, School of Advanced Sciences, Vellore Institute of Technology, Chennai, India; 2 School of Computer Science and Engineering, Vellore Institute of Technology, Chennai, India

**Keywords:** entropy measure, HSV color space, Interval-Valued Intuitionistic Fuzzy Set, low-light enhancement, Reptile Search Algorithm

## Abstract

Superiority of images in low light is necessary in the case of medical image as well as autonomous systems but there is still a challenge of balancing between brightness and natural appearance. The presented paper elaborates a new improvement model that combines Interval-Valued Intuitionistic Fuzzy Set as well as Reptile Search Algorithm optimization. The proposed approach automatically tunes the fuzzy membership and hesitation factors to adapt to uncertainty in dark areas while preserving significant structural data. The Performance is evaluated using common objective metrics which are Peak Signal-to-Noise Ratio, Absolute Mean Brightness Error, Contrast Improvement Index and entropy. All the reported percentage improvements are computed using the average metric values of the baseline Interval-Valued Intuitionistic Fuzzy Set method on the complete dataset. The results of the investigations indicate significant and consistent increases in the experimental results with a 3.69% percentage gain in entropy, a 21.71% percentage gain in brightness restoration, an 18.73% percentage gain in contrast and a 66.12% percentage gain in Peak Signal to Noise Ratio compared to the baseline method. As these results show, the given technique yields naturally amplified images that have better qualities in clarity, conciseness and structural conservation, which is extremely applicable in real-life situations involving low-light photography.

## 1 Introduction

The image processing of digital images is a major feature in various intelligent systems such as medical imaging, surveillance, autonomous driving and facial recognition (Zhao et al., 2019; Mohammed and Al-Tuwaijari, 2022; Badue et al., 2021). The clarity and quality of the input images is of great importance to the performance of these applications. In real-life scenarios, though they usually get shot in underprivileged or unreliable conditions because of low light, environmental interference, camera constraints and other things in the atmosphere. These are the factors that may add noise, decrease contrast and blur significant details. The low-light situations are particularly problematic since they greatly decrease the visibility and influence on the human perception and precision of the computer vision algorithms. It is due to this fact that low-light image enhancement has been added to the list of preprocessing operations, intended to assist in enhancing brightness, restoring crucial data and give rise to naturally looking results. The complexity of such a balance development has stimulated the creation of approaches, including traditional enhancement methods, more sophisticated optimization-oriented and learning-oriented systems.

Image enhancement methods have widely been categorized under spatial-domain and frequency-domain in terms of their use over the years. The most basic technique is called Histogram Equalization (HE) and this method enhances contrast by reallocating intensity levels. Although, HE tends to bring undue brightness changes and artifacts particularly in the high-intensity areas. As a remedy to these problems, Pizer (1977) proposed the Adaptive Histogram Equalization (AHE) that enhances the visibility of the local details by applying the equalization in smaller image tiles, but often increased noise in the smooth areas. Haddadi et al. (2023) optimized the conventional Adaptive Histogram Equalization into an optimized Contrast Limited Adaptive Histogram Equalization (CLAHE), which controls the levels up or down of contrast utilization by using a clipping threshold and an optimization mechanism that enhances the quality of images at the lowest feasible noise levels. Other than methods on histograms, there are color space transformations, which have been applied to improve images. Kumar and Jindal (2019) combined the RGB and HSV channels and minimized the haze of foggy image, without damaging structural information and contrast. Wu et al. (2024) also use the V(brightness) channel of HSV to improve images without altering hue and saturation to ensure that the images retain the soft, natural colors.

Although the traditional practices seem to offer some relevant improvement, their lack of flexibility to confront rough image conditions and the inability to control noise usually introduced qualitatively unnatural effects. It is this failure that has encouraged researchers to look at deep learning techniques, which have recently taken the limelight in providing a more flexible, robust and natural enhancement. Wei et al. (2018) presented Retinex-Net that separates pictures into components of illumination and reflectance, which works with the adjustment of illumination and then suppresses noise in the reflectance component and reconstructs the final results with a multi-scale method. Jiang et al. (2021) proposed EnlightenGAN that uses an unsupervised setup of a generative adversarial model where illumination changes are learned directly off of the data and that visually natural results are generated without the use of paired data. On the same note, Zero-DCE presented by Guo et al. (2020) developed enhancement into a curve estimation problem by which the authors designed a small network that can enhance in real-time with zero-reference learning. Elaborating on them, Yang et al. (2023) proposed LightenNet, a convolutional architecture that can directly learn the brightness mapping functions that allow enhancing low-light scenes adaptively. FlightNet is a proposal of Ozcan et al. (2023)) that is a lightweight architecture tested with the aim of maintaining a balance between computational efficiency and high quality improvement where it is fine-tuned to operating real-time in devices with low resources. The SCI as developed by Ma et al. (2022) improves the images through the learning of illumination adjustment in the self-supervised mode by removing the requirement of paired datasets and maximizing flexibility. Since also, more recently, Demir and Kaplan (2023) introduced SSIF that combines both spatial and spectral information to improve the visibility and reveal the fine detail in the complicated low-light scenarios. Moreover, transformer models, including Uformer suggested by Wang et al. (2022), has demonstrated high levels of generalization to restoration tasks, including similarity to low-light enhancement through self-attention to detect relations on both local and global dependencies to improve the images in a specific dataset. The deep learning techniques are thought to be the most common and effective option but the training of such networks would need a performance hardware and the running time can take a long time greatly varying meaning that it would require a long time depending on the network structure. Wang et al. (2020) conducted an experimental review of the traditional methods, including the Retinex, machine learning methods, frequency-based, which have offered a comprehensive examination of the benefits and shortcomings of every approach.

Through these developments, fuzzy set theory has become widely used in dealing with uncertainty in the digital image processing. The images in digital form tend to leave uncertainty because of the clarity and the general quality of the image. This uncertainty exists in the case of low-light image enhancement, which involves the difference in brightness and darkness between the various parts of an image. Fuzzy set theory was introduced by Zadeh (1965), which is successfully addresses this issue by dealing with the fuzziness in intensity levels. So, thereby improving image quality through enhanced contrast. In fuzzy set-based image enhancement, the degree of contrast level of pixels is represented using membership functions. For instance, Singh et al. (2021) have created a fuzzy-based image enhancement method, which involves using modified membership helps to enhance the global contrast and maintain color image visual quality. Liu et al. (2022) came up with a straight forward, yet effective improvement technique that integrates an adaptive membership function with gamma correction to improve brightness and reduce uneven illumination. But traditional fuzzy sets just take into consideration a membership degrees and explicitly report nothing on hesitation or uncertainty in the assignment of values. In order to eliminate this problem, Atanassov (1999) generalized fuzzy sets into Intuitionistic Fuzzy Sets (IFS) with a hesitation component in addition to membership function. This extension is enhanced better than fuzzy sets, In order to create the hesitation element in IFS, Chaira (2020) developed the Intuitionistic Fuzzy Generator (IFG) and it was inspired by Yager and Sugeno generating functions, to construct an IFS from a fuzzy set. The IFG involves a parameter that determines the final IFS representation with different parameter values used for different applications. For example, Chaira (2011) tested a parameter of 0.85 when coming up with an intuitionistic fuzzy c -means clustering algorithm of CT brain image segmentation, and subsequently used a constant parameter of 0.1 in low-contrast mammogram image enhancement (Chaira, 2020).

Building on this concept, Jebadass and Balasubramaniam (2022b) proposed a technique to enhance low-light images via IFS, where Yager's IFG was employed to build Intuitionistic Fuzzy Image (IFI). This parameter was confined within the range of 0.1–1.0 in steps of 0.1 to generate ten virtual images with the best being selected based on the highest entropy to be the final output image optimized. Extending this work further, Jebadass and Balasubramaniam (2022a) proposed Interval-Valued Intuitionistic Fuzzy Set (IVIFS) to improve image quality as well with the help of the generating function of Yager. The parameter in this case was set between 0.1 and 1.0 with a step size of 0.1 giving a total output of 100 candidate images with the one having maximum entropy being selected. Subsequently, in “Color Image Enhancement Technique Based on IVIFS” Jebadass and Balasubramaniam (2024) allow parameter values to change between 0.1 to 10 as the generating function of Chaira. Such were combined with the hesitation image and added to the membership image to create the Interval-Valued intuitionistic fuzzy image (IVIFI) that produced 1,000 candidate images, one of which was chosen as the best one in terms of entropy. Jebadass and Balasubramaniam (2023) presented the “Interval Type-2 Fuzzy Set Based Block-SBU for Image Fusion Technique,” based image fusion method based on the generating function of Chaira. The IFI in this approach was changed into interval type-2 fuzzy images and that was followed with block separation, unification and successful fusion and conversion of blur images by varying values of β and ζ starting with 0.1 expressed in multiples of 0.1 as a fuzzy-based image fusion approach using Chaira's generating function. The new intuitionistic fuzzy generator (IFG) is suggested by Selvam et al. (2024) to build IFI, in which CLAHE was used instead of histogram equalization (HE). the parameter range ranging between 0.1 to 1.0 with the image of the highest entropy being selected. This chain of research was continued further in Selvam and Sundaram (2025) by use of IVIFS where Chaira generating function was applied to movements of parameter variation ranging between 0.1 to 1.0 with the orchestration of steps as 1.0. The methodology in contrast to the previous ones to determine the best image based on the best entropy index, picks the image with the best structural similarity index (SSIM) index. On the same note, Ragavendirane and Dhanasekar (2025) provided an improvement process, though the parameter was set to be between 0 and 1.0 in intervals of 0.01, producing 100 candidate images and the image with the greatest entropy as the enhanced output. A new intuitionistic fuzzy generator of low-light video-enhancement was proposed by Chinnappan and Sundaram (2024) by employing 0.1–1.0 as the parameters, as ten images were created per frame, and the frame with the highest level of entropy was enhanced with histogram equalization. This showed enhancement in the contrast, less noise and higher structural similarity over current methods. Later doing the same work, Chinnappan and Sundaram (2025) suggested using an image optimization strategy that uses both IVIFS-based fuzzification with a fractional Sobel operator to enhance edges and details. Parameters ζ (0.1–δ) and the degree of hesitation ${\tilde{J}}_{M}$ (0.1–ρ) were used in order to produce a number of candidate images with the optimal image selected depending on the entropy. Such a technique guaranteed the best contrast, retention of detail and likeness of form.

Based on this literature, it can be seen that parameter values are usually taken as discrete fixed sets as opposed to optimizing over continuous interpolation of the variables. Despite promising results shown by fuzzy set, IFS and IVIFS-based approaches, they have remained promising. One of the key weaknesses is that they are based on brute-force adjustment of parameters. In most existing solutions, a large number of candidate images are created by changing the parameters with discrete variations (e.g., 0.1–10) after which the one with the largest entropy or SSIM is chosen. Although it is a tradeoff between reasonable improvement and computational cost, and is not adaptive because similar range of parameters might not be the best for every image. Also, discrete sampling can fail to achieve the optimal point in the interval causing suboptimal improvement. Such constraints encourage the creation of optimization based techniques that can find the most appropriate parameter values in a dynamic manner to enhance them effectively and successfully.

To overcome this drawback, a number of scholars have integrated the concept of optimization in fuzzy-based improvement mechanisms. Kabir et al. (2023) used metaheuristics to fine-tune parameters of the enhancement of the contrast images, whereas Haribabu and Guruviah (2025) suggested an approach called Fermatean fuzzy set and Whale Optimization as FFSWOA Fuse to multimodal fusion of medical images. Rajasekar et al. (2022) created an optimized version of the fuzzy genetic algorithm of multimodal biometric recognition and Dounis et al. (2023) used the state-of-the-art fuzzy sets along with genetic algorithms to enhance the mammographic image quality. Such studies express the usefulness of optimization in enhancing fuzzy-based techniques but it may still be desirable to enhance fuzzy-based approaches in order to guarantee flexibility, prevent early convergence and versatility of various image classes. Although such old methods of optimization like Genetic Algorithm (GA) (Alhijawi and Awajan, 2024), Harmony Search (HS) (Abualigah et al., 2020), Cuckoo Search (CS) (Al-Abaji, 2021), Gray Wolf Optimizer (GWO) (Dada et al., 2022), Krill Herd Algorithm (KHA) (Bhatti et al., 2024), Artificial Bee Colony (ABC) (Pham et al., 2024), and Aquila Optimizer (AO) (Abualigah et al., 2021) but no single algorithm is universally optimal across all problem domains, as stated by the No-Free-Lunch (NFL) theorem (Wolpert and Macready, 2002). It has resulted in a continuous development of some new problem-specific metaheuristic strategies. Based on these observations, the paper will develop a brand new framework based on the low-light image enhancement a system that combines Interval-Valued Intuitionistic Fuzzy Sets (IVIFS) with the Reptile Search Algorithm (RSA). In contrast to traditional methods based on the brute-force or discrete parameter sampling, RSA provides continuous optimization of the fuzzy membership and degree of hesitation to guarantee the flexibility in a variety of imaging situations. In the proposed framework, membership, non-membership and hesitation degrees along with IVIFS domain are used to represent the image in the first stage and actually the uncertainty in the low-illuminated regions is well represented. The well-known idea of the offered method is to optimize these parameters with the help of RSA and maximize a fitness value according to entropy, contrast and brightness to enhance the visual value and dynamic range of the images produced in low-light conditions.

This manuscript has the following structure: Section 2 presents the required background and preliminaries. Section 3 describes the proposed RSA–IVIFS enhancement methodology. Section 4 reports the experimental analysis and results, while Section 5 discusses the limitations and potential applications. Finally, the conclusion of the study is done in Section 6.

## 2 Fundamental concepts

In this section, the author describes the main ideas that are the foundation of the current work and help to have a clearer idea of the suggested methodology.

### 2.1 Color space conversion

In case of illumination-based improvement, the input images are firstly transformed in the HSV color model out of the RGB space. The HSV model separates the chromatic constituents (hue and saturation) of a representation, unlike the RGB model, which makes it especially appropriate in the adjustment of brightness and contrast. In the present work, the enhancement procedure is confined to the V channel since it directly encodes the intensity distribution of the image, while the H and S channels are retained to ensure that the original color fidelity is preserved. The conversion from RGB to HSV is performed using the standard transformation equations reported in Wu et al. (2024).

### 2.2 Fuzzy set and its representation in image

Zadeh (1965) introduced the concept of a fuzzy set to represent vagueness and imprecision. A Fuzzy Set (FS) *A* in a universe of discourse *X* is defined as:


$$\begin{array}{l} A=\left\{\left(x,\mu_{A}\left(x\right)\right)|x\in X\right\}, \end{array}$$ (1)

where μ_*A*_(*x*):*X* → [0, 1] is the membership function that assigns to each element *x* a degree of membership in *A*.

This concept is used in image processing wherein a sharp image, the pixel values of which are usually in the range of 0 to 255, is transformed into a fuzzy image representation. This is accomplished by the use of a membership operation which normalizes the gray levels of the pixels within the range [0, 1]. The fuzzification process of an image *S* is written as.


$$\begin{array}{l} \mu_{ij}=\frac{\gamma_{ij}-\gamma_{\min}}{\gamma_{\max}-\gamma_{\min}}, \end{array}$$ (2)

where γ_*ij*_ is the value of the intensity of a pixel, γ_min_ and γ_max_ are the maximum and minimum gray levels of the image respectively. This fuzzy representation provides a foundation for extending classical fuzzy concepts are broadened to a higher level of representation like IFS and IVIFS.

### 2.3 Intuitionistic Fuzzy Sets (IFSs)

The Intuitionistic Fuzzy Sets (IFSs) are a category of fuzzy sets constructed based on the intuitionistic logic instead of a classical or traditional logic approach. This sub section is a summary of the IFSs. It brings in the noteworthy mathematical constructs and provides the procedure involved in developing an IFS and creating the relatable Intuitionistic Fuzzy Image (IFI).

#### 2.3.1 Mathematical foundation of IFSs

To generate membership and non-membership functions, Atanassov (1999) presented an improved fuzzy set variant known as the IFS. An IFS on a universe of discourse *X* can be described as


$$\begin{array}{l} F=\left\{\left(x,\mu_{F}\left(x\right),\nu_{F}\left(x\right)\right)|x\in X\right\}, \end{array}$$ (3)

where μ_*F*_(*x*):*X* → [0, 1] and ν_*F*_(*x*):*X* → [0, 1] denote the membership and non-membership degrees of an element *x* with the condition 0 ≤ μ_*F*_(*x*) + ν_*F*_(*x*) ≤ 1 and also the hesitancy degree π_*F*_(*x*) is defined as π_*F*_(*x*) = 1 − μ_*F*_(*x*) − ν_*F*_(*x*), where π_*F*_(*x*) ∈ [0, 1]. Thus, for every x∈X, the relation μ_*F*_(*x*) + ν_*F*_(*x*) + π_*F*_(*x*) = 1 is satisfied.

#### 2.3.2 Construction of IFS and Intuitionistic Fuzzy Image (IFI)

In the previous section, the mathematical background of the IFS was discussed, where each pixel in the image is represented by a membership and non-membership degree. However, to practically apply this concept in image enhancement, it becomes important to construct suitable functions that can generate these values accurately for each pixel. To achieve this, an Intuitionistic Fuzzy Generator (IFG) plays a significant role in extending fuzzy sets into IFS and is defined as a function


$$\begin{array}{l} \psi :\left[0,1\right]\to \left[0,1\right] \end{array}$$

that satisfies the condition


$$\begin{array}{l} \psi \left(x\right)\leq \left(1-x\right),\forall x\in \left[0,1\right]. \end{array}$$ (4)

The development of an Intuitionistic Fuzzy Generator (IFG) begins with characterizing a fuzzy complementary function, which can be realized by selecting either an increasing or a decreasing function. In this work, an IFG is developed using Chaira (2020) generating function to effectively handle low-light images.

Let *n*:[0, 1] → [0, 1] be an increasing function that satisfies the condition of an involutive fuzzy complement. A function *n*(*x*) is said to be an involutive fuzzy complement if and only if there exists a continuous, strictly increasing function *L* such that *L*(0) = 0. In this case, the fuzzy complement is expressed as:


$$\begin{array}{l} n\left(\mu \left(x\right)\right)=L^{-1}\left(L\left(1\right)-L\left(\mu \left(x\right)\right)\right). \end{array}$$ (5)

To construct the complement, we consider the generating function defined as:


$$\begin{array}{l} L\left(x\right)=\frac{1}{a}\log\left[1+x\left(1+a\right)\right], \end{array}$$ (6)

where *a* is a control parameter that adjusts the shape of the fuzzy transformation curve. Substituting *x* = 0 and *x* = 1 in (6) :


$$\begin{array}{l} L\left(0\right)=\frac{1}{a}\log\left(1\right)=0,\;L\left(1\right)=\frac{1}{a}\log\left(2+a\right). \end{array}$$

The inverse of *L*(*x*) can be expressed as:


$$\begin{array}{l} L^{-1}\left(x\right)=\frac{e^{ax}-1}{1+a}. \end{array}$$ (7)

By substituting *L*(*x*) and *L*^−1^(*x*) into the (5), the fuzzy complement function becomes:


$$\begin{array}{l} n\left(x\right)=L^{-1}\left[\frac{1}{a}\log\left(2+a\right)-\frac{1}{a}\log\left(1+\left(1+a\right)x\right)\right], \end{array}$$ (8)

$$\begin{array}{l} =L^{-1}\left[\frac{1}{a}\log\left(\frac{2+a}{1+\left(1+a\right)x}\right)\right]. \end{array}$$

Now, using (7) in (9) we get,


$$\begin{array}{l} n\left(x\right)=\frac{e^{\log\left(\frac{2+a}{1+\left(1+a\right)x}\right)}-1}{1+a}, \end{array}$$ (9)

$$\begin{array}{l} n\left(x\right)=\frac{\frac{2+a}{1+\left(1+a\right)x}-1}{1+a}, \end{array}$$

$$\begin{array}{l} n\left(x\right)=\frac{\left(2+a\right)-\left[1+\left(1+a\right)x\right]}{\left(1+a\right)\left[1+\left(1+a\right)x\right]}, \end{array}$$

$$\begin{array}{l} =\frac{1-x}{1+\left(1+a\right)x},\;a>1. \end{array}$$ (10)

Thus, the fuzzy complement is expressed as:


$$\begin{array}{l} n\left(x\right)=\frac{1-x}{1+\left(1+a\right)x}. \end{array}$$ (11)

Based on ψ(*x*) in (4), the corresponding non-membership function is defined as:


$$\begin{array}{l} n\left(\mu \left(x\right)\right)=\psi \left(\mu \left(x\right)\right)=\frac{1-\mu \left(x\right)}{1+\left(1+a\right)\mu \left(x\right)},\;a>0. \end{array}$$ (12)

The complement relation is employed to compute the enhanced membership value $\mu_{ij}^{\prime }$ at pixel position (*i, j*), given by:


$$\begin{array}{l} \mu_{ij}^{\prime }=1-n\left(\mu_{ij}\right). \end{array}$$ (13)

Substituting (12) in (13) gives


$$\begin{array}{l} \mu_{ij}^{\prime }=1-\frac{1-\mu_{ij}}{1+\left(1+a\right)\mu_{ij}}, \end{array}$$

$$\begin{array}{l} \mu_{ij}^{\prime }=\frac{\left(a+2\right)\mu_{ij}}{1+\left(a+1\right)\mu_{ij}}. \end{array}$$ (14)

This illustrates the enhanced membership function for the image.

Similarly, the non-membership function for the enhanced image is obtained by using (12) as


$$\begin{array}{l} \nu_{ij}^{\prime }=\psi \left(\mu_{ij}^{\prime }\right)=\frac{1-\mu_{ij}^{\prime }}{1+\left(1+a\right)\mu_{ij}^{\prime }}. \end{array}$$ (15)

Substituting (14) in (15) we get,


$$\begin{array}{l} \nu_{ij}^{\prime }=\frac{1-\mu_{ij}}{1+\left(a^{2}+a+3\right)\mu_{ij}}. \end{array}$$ (16)

Finally, the hesitation value for the image at each pixel is computed using the standard intuitionistic fuzzy relation:


$$\begin{array}{l} \pi_{ij}^{\prime }=1-\mu_{ij}^{\prime }-\nu_{ij}^{\prime }. \end{array}$$ (17)

### 2.4 Interval-Valued Intuitionistic Fuzzy Sets (IVIFIs)

This subsection represent the overview of IVIFSs. It outlines the fundamental mathematical ideas and explains how an IVIFS is constructed and subsequently used IVIFI.

#### 2.4.1 Mathematical foundation of IVIFIs

An IVIFS F^*^ on a universe of discourse *X* is defined as


$$\begin{array}{l} F^{*}=\left\{\left(x,M_{F^{*}}\left(x\right),N_{F^{*}}\left(x\right)\right)|x\in X\right\}, \end{array}$$ (18)

where $M_{F^{*}}\left(x\right)\subseteq \left[0,1\right]$ and $N_{F^{*}}\left(x\right)\subseteq \left[0,1\right]$ denote the membership interval and the non-membership interval of the element *x* ∈ *X*. These intervals are represented as


$$M_{F^{*}}\left(x\right)=[\inf M_{F^{*}}\left(x\right),\sup M_{F^{*}}\left(x\right)],\;N_{F^{*}}\left(x\right)=[\inf N_{F^{*}}\left(x\right),\sup N_{F^{*}}\left(x\right)],$$

and it must satisfy the condition $\sup M_{F^{*}}\left(x\right)+\sup N_{F^{*}}\left(x\right)\leq 1,\forall x\in X.$

#### 2.4.2 Construction of IVIFS

Although IFS effectively reduces ambiguities in images, practical scenarios such as sensor noise and unclear boundaries provide a level of uncertainty that remains challenging to quantify. The hesitation degree in IFS being a single, definitive number, may not adequately represent this variability. To address this issue, IVIFS was first introduced by Bustince and Burillo (1995), extend IFS by representing membership and non-membership as intervals rather than fixed values.

Now to construct the IVIFS by defining a mapping as follows:


σ:IFS→IVIFS

$$\begin{array}{l} \sigma \left(F\right)=\{\left(x,M_{\sigma \left(F\right)},N_{\sigma \left(F\right)}|x\in X\right\}=F^{*}, \end{array}$$ (19)

*M*_σ(*F*)_ and *N*_σ(*F*)_ are split into the upper and lower limits of the membership and non membership intuitionistic fuzzy intervals, respectively. These components are defined as follows:

1.
$M_{\sigma \left(F\right)U}\left(x\right)=M_{F^{*}Ũ}\left(x\right)=\mu_{ij}^{\prime }\left(x\right)+\omega \cdot \pi_{ij}^{\prime }\left(x\right),\;0\leq \omega \leq 1.$

2.
$M_{\sigma \left(F\right)L}\left(x\right)=M_{F^{*}\tilde{L}}\left(x\right)=\mu_{ij}^{\prime }\left(x\right)-c\cdot \pi_{ij}^{\prime }\left(x\right),\;0\leq c\leq \frac{\mu_{ij}^{\prime }\left(x\right)}{\pi_{ij}^{\prime }\left(x\right)}.$

3.
$N_{\sigma \left(F\right)U}\left(x\right)=N_{F^{*}Ũ}\left(x\right)=\nu_{ij}^{\prime }\left(x\right)+\rho \cdot \pi_{ij}^{\prime }\left(x\right),\;0\leq \rho \leq 1.$

4.
$N_{\sigma \left(F\right)L}\left(m\right)=N_{F^{*}\tilde{L}}\left(x\right)=\nu_{ij}^{\prime }\left(x\right)-d\cdot \pi_{ij}^{\prime }\left(x\right),\;0\leq d\leq \frac{\nu_{ij}^{\prime }\left(x\right)}{\pi_{ij}^{\prime }\left(x\right)}.$

The parameters ω, *c*, ρ, *d* weight the influence of the hesitation degree on the interval bounds. Specifically, ω and *c* adjust the membership interval, while ρ and *d* adjust the non-membership interval. With the constraints 0 ≤ ω+ρ ≤ 1, 0 < ω+*c* ≤ 1, 0 < ρ+*d* ≤ 1.

Next, the interval widths can be expressed as


$$\begin{array}{l} h_{M}=M_{F^{*}Ũ}\left(x\right)-M_{F^{*}\tilde{L}}\left(x\right)=\left(\omega +c\right)\cdot \pi_{ij}^{\prime }\left(x\right)=\alpha_{M}\cdot \pi_{ij}^{\prime }\left(x\right), \end{array}$$ (20)

$$\begin{array}{l} h_{N}=N_{F^{*}Ũ}\left(x\right)-N_{F^{*}\tilde{L}}\left(x\right)=\left(\rho +d\right)\cdot \pi_{ij}^{\prime }\left(x\right)=\beta_{N}\cdot \pi_{ij}^{\prime }\left(x\right). \end{array}$$ (21)

Where α_*M*_ and β_*N*_ correspond to (ω+*c*) and (ρ+*d*), respectively.

This construction confirms the gap between membership and non-membership values in IVIFS never exceeds the intuitionistic fuzzy index $\left(\pi_{ij}^{\prime }\right)$. Here, if *F* ∈ *FS*, then the mapping is defined as


$$\begin{array}{l} \sigma \left(F\right)=\left\{\left(x,M_{\sigma \left(F^{*}\right)},N_{\sigma \left(F^{*}\right)}|x\in X\right)\right\} \end{array}$$

with $\pi_{ij}^{\prime }\left(x\right)=0$, implies


$$\begin{array}{l} M_{F^{*}Ũ}\left(x\right)=M_{F^{*}\tilde{L}}\left(x\right)=\mu_{ij}^{\prime }\left(x\right), \end{array}$$ (22)

$$\begin{array}{l} N_{F^{*}Ũ}\left(x\right)=N_{F^{*}\tilde{L}}\left(x\right)=\nu_{ij}^{\prime }\left(x\right), \end{array}$$ (23)

which implies σ(*F*) = *F*. Therefore, if *F* ∈ *FS* then σ(*F*) = *F*.

#### 2.4.3 Interval-Valued Intuitionistic Fuzzy Image (IVIFI)

The low-light input image *S* = [γ_*ij*_] of size *k* × *l*, is normalized using (2), and the resulting values are used to create a fuzzified image. During this fuzzification step assigns a membership value to each pixel, improving low-intensity information and maintaining the structural details. The fuzzified image is then converted into an Intuitionistic Fuzzy Image (IFI) *F* = [*f*_*ij*_] by applying the transformation in (14), which incorporates both membership and non-membership values to better model pixel uncertainty. To further enrich this representation, the IFI is extended to an Interval-Valued Intuitionistic Fuzzy Image (IVIFI) by combining the transformations in (14) and (20). This extension defines interval limits that are determined by the hesitation value $\pi_{ij}^{\prime }$, resulting in an IVIFS representation $F^{*}=\left[f_{ij}^{*}\right]$ defined in (24).


$$\begin{array}{l} f_{ij}^{*}=\mu_{ij}^{\prime }+\left(\omega +c\right)\pi_{ij}^{\prime }, \end{array}$$

$$\begin{array}{l} f_{ij}^{*}=\mu_{ij}^{\prime }+h_{M}. \end{array}$$ (24)

Here, $\mu_{ij}^{\prime }$ represents the modified membership degree, while the parameters ω and *c* determine how the hesitation term adjusts the membership interval. Unlike the IFI, which assigns a single value to each component, the IVIFI captures pixel uncertainty using intervals, providing more flexibility in representing ambiguous regions and improving the preservation of structural details in low-light images.

## 3 Proposed method

**Figure 1:**
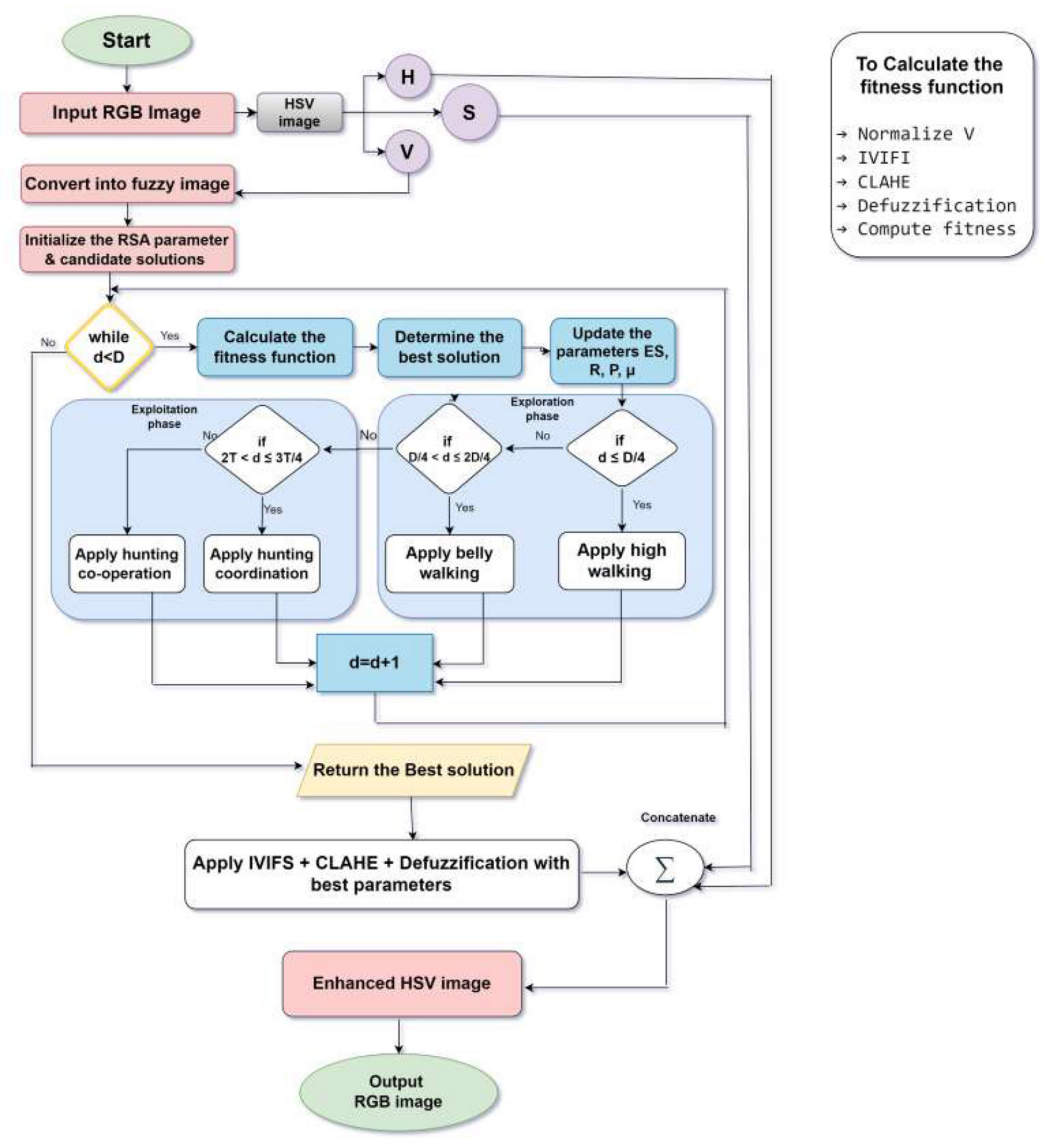
Flowchart demonstrate the proposed IVIFS-RSA enhancement framework.

In this section, the proposed methodology of RSA-IVIFI enhancement is described, where image improvement as the Reptile Search Algorithm and an optimization problem is used to create the most optimal IVIFI parameters. The general design has three major aspects: the problem formulation, the RSA optimization strategy, and the complete RSA-IVIFI enhancement algorithm. Figure 1 illustrates the full process working diagram of the proposed RSA-IVIFI improvement algorithm. The conversion of the image starts the process based on conversion of RGB to HSV (Section 2.1), the luminance information is isolated in the V-channel while preserving color components in H and S channels. The fundamental improvement process employs the RSA (Section 3.2) to optimize IVIFI parameters (*a*, α_*m*_) through an iterative optimization loop. This optimization is driven of four behavioral states of the RSA: High Walking for global exploration, Belly Walking for local search, Hunting Coordination for collaborative information sharing, and Hunting Cooperation for final convergence. These steps, as seen in Figure 1 are mathematically reasoned out in Sections 3.2.2–3.2.5. After each candidate solution goes through the fitness function (Section 3.1) it is computed to estimate the quality of each solution after it undergoes the complete enhancement pipeline: IVIFI transformation (Section 2.4), CLAHE processing (Section 3.4), and defuzzification (Section3.5). The detailed mathematical formulation of the proposed methodology is described in the upcoming subsections.

### 3.1 Problem formulation

Images taken in low-light are usually full of low contrast, low brightness and structural detail. Consequently, the process of improvement is developed into an optimization problem in order to discover it automatically. The optimum values of the IVIFS-based visual enhancement approach which optimize the visuals of the resulting images. In this paper, the proposed fitness function is formulated as a multi-objective function. To balance these objectives, a normalized weight coefficients of *w*_1_ = 0.4, *w*_2_ = 0.3 and *w*_3_ = 0.3 are assigned, where the slightly higher value of *w*_1_ emphasizes entropy to preserve fine details, while *w*_2_ and *w*_3_ ensure balanced contrast and brightness retention. The fitness function proposed in (Acharya and Kumar, 2021) employs equal weighting for all components, which may optimize statistical measures but often compromises perceptual quality. In contrast, the proposed fitness function in this work is designed to prioritize visual quality and detail preservation, producing enhanced images that are both statistically consistent and perceptually superior.

The first objective function is represented as entropy, which evaluates the amount of information content present in the enhanced image. It is mathematically represented as


$$\begin{array}{l} \hat{E}\left(I_{\text{enh}}\right)=-\overset{L-1}{\underset{i=0}{\sum }}p_{i}\log_{2}\left(p_{i}\right). \end{array}$$ (25)

where *p*_*i*_ is the normalized histogram probability of gray level *i* and *L* represents the total number of intensity levels. An increased value of entropy implies that the pixel intensities have a more even distribution, denotes that the improvement procedure has been effective in uncovering concealed data and finer structural. some details that were not seen in the low-light original image.

The second objective role is determined by Contrast Improvement Index (CII) which determines the assessment of the increased contrast between the original image *I*_*o*_ and enhanced image *I*_enh_. It is formulated as


$$\begin{array}{l} \hat{CII}\left(I_{o},I_{\text{enh}}\right)=\frac{C\left(I_{\text{enh}}\right)}{C\left(I_{o}\right)}, \end{array}$$ (26)

where


$$\begin{array}{l} C\left(I\right)=\sqrt{\frac{1}{MN}\overset{M}{\underset{x=1}{\sum }}\overset{N}{\underset{y=1}{\sum }}{\left(I\left(x,y\right)-\mu_{I}\right)}^{2}}. \end{array}$$ (27)

Here, the standard deviation–based contrast measure of an image *I*, *M* × *N* is the image size, and μ_*I*_ is the mean intensity. A value of *CII* > 1 indicates that the enhanced image displays improved contrast than the original, indicating successful enhancement and improved edges clarity and fine textures.

The third objective function considers brightness preservation, which is evaluated using the Absolute Mean Brightness Error (AMBE). It ensures that the enhancement process maintains the overall natural brightness level of the original image and avoids over-enhancement or darkening and It is formulated as


$$\begin{array}{l} \hat{AMBE}\left(I_{o},I_{\text{enh}}\right)=|M\left(o\right)-M\left(e\right)|. \end{array}$$ (28)

where M(o) and M(e) denote the mean intensities of the original and enhanced images, respectively.

By combining these three objective functions, the overall fitness function is formulated as


$$\begin{array}{l} \underset{a^{*},h_{M}^{*}}{\max}F\left(a^{*},h_{M}^{*}\right)=0.4\hat{E}\left(I_{\text{enh}}\right)+0.3\hat{CII}\left(I_{o},I_{\text{enh}}\right)+0.3\hat{AMBE}\left(I_{o},I_{\text{enh}}\right). \end{array}$$ (29)

where the parameters *a* and *h*_*M*_ act as the control variables of the IVIFS transformation that determine the amount of hesitation, fuzzification and scaling during enhancement. The goal of the optimization is to determine the optimal parameter set $\left[a^{*},h_{M}^{*}\right]$ that yields the highest fitness value *F*(*a, h*_*M*_), resulting in an enhanced image with higher improved contrast, information content, and preserved natural brightness. The parameters are optimized within bounded ranges, with *a* ∈ [0.1, 10] and *h*_*M*_ ∈ [0.1, 1], which define the feasible search space of the optimization problem.

### 3.2 Reptile search algorithm

This section demonstrates the optimization task of determining the most effective parameters (*a, h*_*M*_) for enhancing the contrast of low-light images within the Interval-Valued Intuitionistic Fuzzy Set (IVIFS) and CLAHE framework. The Reptile Search Algorithm (RSA) was adopted as the optimization method based on its advantages over other metaheuristics for this type of problem domain. The optimization problem in IVIFS-CLAHE presents three major challenges: (1) the requirement for precise fine-tuning of enhancement parameters (2) a multimodal fitness surface with multiple local optima, and (3) complex interdependencies among contrast, brightness preservation and structural integrity metrics. RSA effectively addresses these challenges due to its adaptive design and balanced search dynamics.

Unlike Genetic Algorithms (GA), in where disruptive crossover operations can slow down convergence, or Particle Swarm Optimization (PSO) that is associated with premature convergence in search complexities spaces, RSA uses a two stage process that is well organized, which ensures that there is a distinction between exploration (global search) and exploitation (local search). There are four integrated mathematical models of these strategies: high walking, belly walking, coordination of hunting and cooperation in hunting. The exploratory the phase retains a diverse population to cover the globe effectively whereas the exploitative phase executes its functions and solutions with high accuracy an important feature to control exactly the fine parameters (*a, h*_*M*_) in CLAHE. In addition to its biological motivation, the mechanisms of RSA have the practical optimization benefits. Crocodiles' exceptional low-light vision and cooperative hunting behaviors reflect RSA's ability to adaptively balance wide-ranging exploration with precise exploitation, supports both robustness and efficient convergence.

Benchmark studies in the foundational RSA literature Abualigah et al. (2022) have demonstrated its improved optimization accuracy, convergence rate and reliable performance compared with GA, PSO and Differential Evolution (DE) across 63 benchmark functions. A recent survey by Sasmal et al. (2024) reported that RSA achieves an efficient balance between exploration and exploitation with competitive computational performance compared to other commonly applied metaheuristics. These findings confirm RSA's suitability for solving highly nonlinear and multimodal optimization problems. Owing to these properties, RSA is well suited for optimizing (*a, h*_*M*_) parameters that boost image contrast and perceptual quality while maintaining the natural appearance of low-light images.

The following subsections describe the three major stages of RSA: Initialization, Exploration and Exploitation.

#### 3.2.1 Initialization phase

In RSA (Abualigah et al., 2022), the optimization process begins with the generation of an initial population of candidate solutions, which can be represented in the form of a matrix *Y*:


$$Y=\left[\begin{array}{ccccc} y_{1,1} & \cdots & y_{1,j} & \cdots & y_{1,𝔫}\\ y_{2,1} & \cdots & y_{2,j} & \cdots & y_{2,𝔫}\\ \vdots & \ddots & \vdots & \ddots & \vdots \\ y_{N,1} & \cdots & y_{N,j} & \cdots & y_{N,𝔫} \end{array}\right].$$

Here, *N* denotes the number of candidate solutions in the population and 𝔫 represents the dimensionality of the optimization problem. The notation *y*_(*i, j*)_ corresponds to the value of the *j*^th^ decision variable in the *i*^th^ candidate solution. The initialization of each element is performed using:


$$\begin{array}{l} y_{\left(i,j\right)}=LB+\text{rand}\times \left(UB-LB\right),\;j=1,2,\ldots ,𝔫. \end{array}$$ (30)

where *UB* and *LB* denote the upper and lower bounds of the search space, respectively and rand is a uniformly distributed random number between 0 and 1. This procedure ensures that the initial population is spread across the entire feasible region of the search space.

#### 3.2.2 Encircling phase (exploration)

The encircling stage of RSA is based on the high-walking and belly-walking of crocodiles movements, and these aid it in searching a wide space and then approaching its prey. This mechanism broadens the search space, promotes the process of exploring a large set of candidate solutions and eliminates the risk of premature convergence. The ability to model this behavior used by RSA puts it in a better position to identify promising areas during the search domain and thus a good foundation to the subsequent stage of later hunting (exploitation).

#### 3.2.3 Strategy 1: high walking

The exploration process enables the reptile to detect the prey area and establish the optimal hunting region using the high walking strategy when *d* ≤ *D*/4. During the exploration phase, the reptile identifies the prey region and determines the optimal hunting zone by adopting the high walking strategy, is formulated as


$$\begin{array}{l} y_{\left(i,j\right)}\left(d+1\right)={\text{Best}}_{j}\left(d\right)\times -\eta_{\left(i,j\right)}\left(d\right)\times \beta -R_{\left(i,j\right)}\left(d\right)\cdot \text{rand},\;d\leq \frac{D}{4} \end{array}$$ (31)

Here, Best_*j*_(*d*) represents the *j*-th position of the best solution obtained up to the current iteration. η_(*i, j*)_(*d*) denotes the hunting operator that influences the search direction. The parameter β controls the accuracy of the search process, while rand is a random number in the range [0, 1] that maintains population diversity. The variable *d* is the current iteration number and *D* is the maximum number of iterations allowed. The term *R*_(*i, j*)_(*d*) progressively reduces the search space over time, expressed as


$$\begin{array}{l} \eta_{\left(i,j\right)}\left(d\right)={\text{Best}}_{j}\left(d\right)\times P_{\left(i,j\right)} \end{array}$$ (32)

$$\begin{array}{l} R_{\left(i,j\right)}\left(d\right)=\frac{{\text{Best}}_{j}\left(d\right)-y_{\left(r_{2},j\right)}}{{\text{Best}}_{j}\left(d\right)+\epsilon } \end{array}$$ (33)

In (31), *P*_(*i, j*)_ denotes the percentage difference between the *j*-th position of the best solution and that of the current solution. The index *r*_2_ is a randomly chosen number within the range [1, *N*] where *N* is the population size and The ϵ is a small constant used to avoid division by zero.

#### 3.2.4 Strategy 2: belly walking

When the prey location is identified and the iteration index falls within the range *D*/4 < *d* ≤ 2*D*/4, the reptile adopts the belly walking strategy to prepare the surrounding area and closely monitor the prey. This behavior is mathematically defined as


$$\begin{array}{l} y_{\left(i,j\right)}\left(d+1\right)={\text{Best}}_{j}\left(d\right)\times y_{\left(r_{1},j\right)}\times ES\left(d\right)\times \text{rand},\;D/4<d\leq 2D/4 \end{array}$$ (34)

The term *ES*(*d*) is a probability factor that regulates the movement magnitude, defined as


$$\begin{array}{l} ES\left(d\right)=2\cdot r_{3}\cdot \left(1-\frac{1}{D}\right), \end{array}$$ (35)

where *r*_3_ is a random number in the range [−1, 1] and *D* denotes the maximum number of iterations. This formulation allows *ES*(*d*) to fluctuate between approximately −2 and 2, providing stochastic adjustment of movement strength. This variability helps to maintain population diversity while refining the search around promising regions, thereby strengthening the exploitation capability of RSA.

#### 3.2.5 Hunting phase (exploitation)

In RSA, the hunting phase is inspired by the natural predatory behavior of crocodiles which primarily depends on coordination and cooperation. Once promising regions are detected during the exploration stage, the algorithm shifts into this phase to intensify the search and identify optimal solutions. This stage incorporates two complementary strategies: hunting coordination and hunting cooperation both designed to direct the search agents toward the global optimum.

#### 3.2.6 Strategy 3: hunting coordination

During this strategy, agents expand the search area in the early iterations (*d* ≤ *D*/2) and slowly shift toward convergence as iterations progress (*d* > *D*/2). Once the prey region is identified, the reptile prepares to attack. This strategy operates within the interval 2*D*/4 < *d* ≤ 3*D*/4, and is mathematically expressed in (36)


$$\begin{array}{l} y_{\left(i,j\right)}\left(d+1\right)={\text{Best}}_{j}\left(d\right)\times P_{\left(i,j\right)}\left(d\right)\times \text{rand},\;2D/4<d\leq 3D/4, \end{array}$$ (36)

$$\begin{array}{l} P_{\left(i,j\right)}=\alpha +\frac{y_{\left(i,j\right)}-M\left(y_{i}\right)}{{\text{Best}}_{j}\left(d\right)\cdot \left(UB_{j}-LB_{j}\right)+\epsilon } \end{array}$$ (37)

Here, α is assigned a value of 0.1 serving as a key parameter that regulates both the sensitivity and the precision of the search across iterations. The term *M*(*y*_*i*_) represents the mean position of the *i*-th solution, it is defined as,


$$\begin{array}{l} M\left(y_{i}\right)=\frac{1}{𝔫}\overset{𝔫}{\underset{j=1}{\sum }}y_{\left(i,j\right)}. \end{array}$$ (38)

This ensures a balance between exploration and exploitation by guiding agents toward the prey while maintaining controlled variability in the search.

#### 3.2.7 Strategy 4: hunting cooperation

Hunting cooperation is applied during the later iterations (3*D*/4 < *d* ≤ *D*) and defined as


$$\begin{array}{l} y_{\left(i,j\right)}\left(d+1\right)={\text{Best}}_{j}\left(d\right)-\eta_{\left(i,j\right)}\left(d\right)\times \epsilon -R_{\left(i,j\right)}\left(d\right)\times \text{rand},\;3D/4<d\leq D. \end{array}$$ (39)

This strategy is motivated by cooperative hunting behavior of crocodiles, where agents exchange information and concentrate on the most promising regions of the search space. This coordination and cooperation help reduce the risk of getting trapped in local optima by preserving solution diversity, while still directing the search process toward the global optimum. To support this mechanism, two stochastic parameters α and β are employed, which play a crucial role in avoiding stagnation, especially during the later stages of optimization. The algorithm terminates when the maximum iterations are reached, convergence is achieved or no improvement occurs over successive iterations.

### 3.3 The RSA-IVIFI enhancement algorithm

Algorithm 1 illustrated the integration of RSA-IVIFI enhancement framework, combining the problem formulation established in Section 3.1 with the Reptile Search Algorithm methodology detailed in Section 3.2.

Algorithm 1 **RSA-IVIFI image enhancement.**

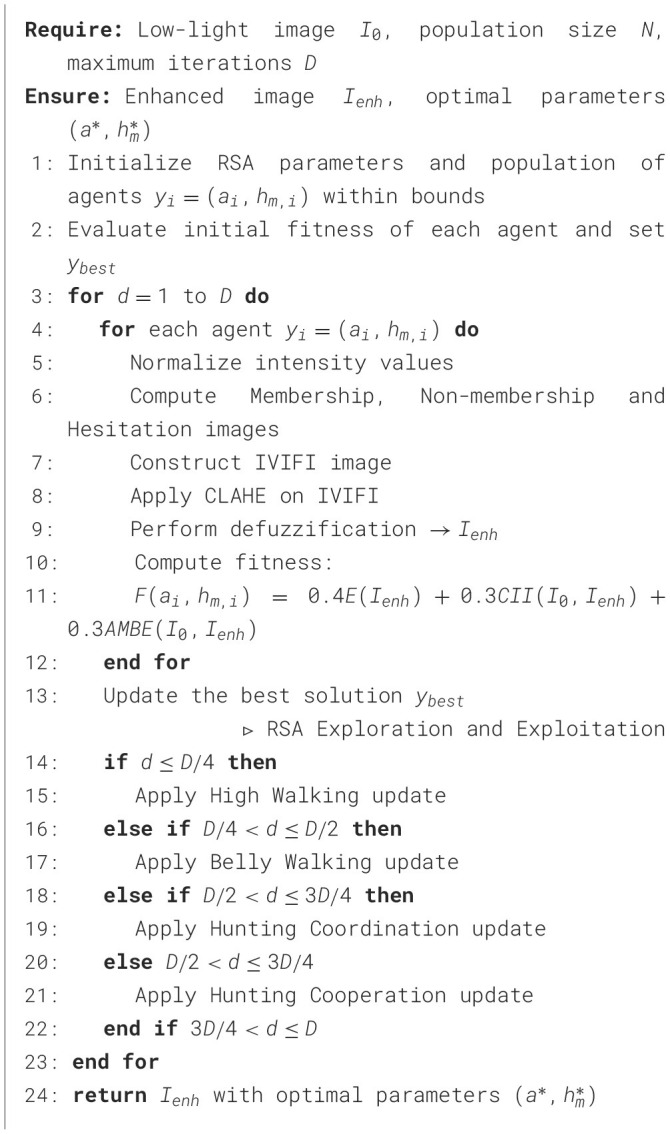

The algorithm requires three inputs: the low-light image *I*_*O*_, the population size *N*, and the maximum number of iterations *D*. The initialization phase generates a population of *N* agents using (30), where each agent *y*_*i*_ corresponds to a candidate solution containing the IVIFI parameters (*a, h*_*M*_) within the defined bounds.

The optimization core consists of an iterative loop executing for *D* cycles, with each iteration comprising three sequential stages:

**Fitness Evaluation:** Each agent's parameters (*a*_*i*_, *h*_*M,i*_) drive the complete IVIFI-CLAHE enhancement pipeline. This process transforms the input image through intensity normalization (2), computation of membership (14), Non membership (16) and hesitation degreee (17) to construct the IVIFI image using (24), CLAHE-based contrast enhancement, and final defuzzification to produce enhanced image candidate *I*_*enh*_. The fitness function *F*(*a*_*i*_, *h*_*M,i*_) from (29) then evaluates enhancement quality through a weighted combination of enhancement measure, contrast improvement index and brightness preservation metric.

**Solution Update:** The algorithm compares all agents' fitness scores and updates the global best solution *y*_*best*_ when superior performance is identified.

**Population Update:** Agent positions are modified using one of four RSA strategies described in Sections [3.2.2 and 3.2.5] and the Strategy selection follows the iterative progression: High Walking (*d* ≤ *D*/4), Belly Walking (*D*/4 < *d* ≤ *D*/2), Hunting Coordination (*D*/2 < *d* ≤ 3*D*/4), and Hunting Cooperation (3*D*/4 < *d* ≤ *D*).

Upon completing *D* iterations, the algorithm returns the enhanced image *I*_*enh*_ generated using the optimal parameters $\left(a^{*},h_{M}^{*}\right)$ derived from the best-performing agent *y*_*best*_.

### 3.4 CLAHE technique and integration of IVIFS-CLAHE

Contrast Limited Adaptive Histization Equalization (CLAHE) improves the local image contrast and at the same time limiting amplification of noise, overcome the weaknesses of traditional histogram equalization. This study uses CLAHE in combination with Interval-Valued Intuitionistic Fuzzy Sets (IVIFS) to maximize low-light image enhancement. The hybrid methodology adheres to known fuzzy-CLAHE methodologies that prove their effectiveness enhanced outcomes of improvement (Padmavathy et al., 2024). The multi-stage system analyses pictures in that order: IVIFS transformation processes the uncertainty of low-light pixel intensities, and then Application of the refined representation to CLAHE. This avoids noise amplification, in addition to optimizing local contrast. In this paper, CLAHE is used on the V cover of the HSV color space, but not H and S channels remain unchanged. The procedure is as follows:

1. Convert the RGB image to HSV and extract the V channel.
2. Divide the V channel into small tiles, for example, 8 × 8 pixels.
3. Compute the histogram for each tile and clip it at a predefined threshold to prevent over-enhancement.
4. Redistribute the clipped pixels uniformly across the histogram bins and apply histogram equalization to enhance the tile.
5. Apply bilinear interpolation across neighboring tiles to remove block boundaries and produce smooth transitions.
6. Merge the enhanced V channel with the original H and S channels and convert the image back to RGB to obtain the final enhanced image.

In this work, CLAHE is integrated with an Interval-Valued Intuitionistic Fuzzy Set (IVIFS) framework to form a sequential enhancement pipeline. The process begins with the IVIFS stage, which is designed to handle the inherent uncertainty in noise reduction and in dark space, resulting in a cleaner intermediate image. This pre-conditioned image is then serves as the input for the CLAHE algorithm. With the noise already mitigated, CLAHE can robustly enhance local contrast, preventing the noise amplification that typically plagues such methods. This combined methodology ensures that the contrast enhancement is applied to a pre-optimized image, leading to more reliable and superior results.

### 3.5 Defuzzification of fuzzy image

Defuzzification converts a fuzzy image into a sharp image, which is suitable for visualization and further processing. The input image in the proposed method is initially converted into fuzzy domain in order to get the fuzzy intensity values. The maximum and minimum gray levels of the original image (*Z*_min_ and *Z*_max_) are determined and each fuzzy pixel *Z*_*mn*_ is mapped to a crisp value $Z_{mn}^{\prime }$ using


$$\begin{array}{l} Z_{mn}^{\prime }=Z_{mn}\cdot \left(Z_{\text{max}}-Z_{\text{min}}\right)+Z_{\text{min}}, \end{array}$$ (40)

producing the resultant improved image. This approach is simple, computationally efficient, preserves the original dynamic range, effectively enhances visibility and contrast in low-light images. Alternative defuzzification strategies can also be employed depending on specific application requirements.

## 4 Experimental analysis

**Figure 2:**
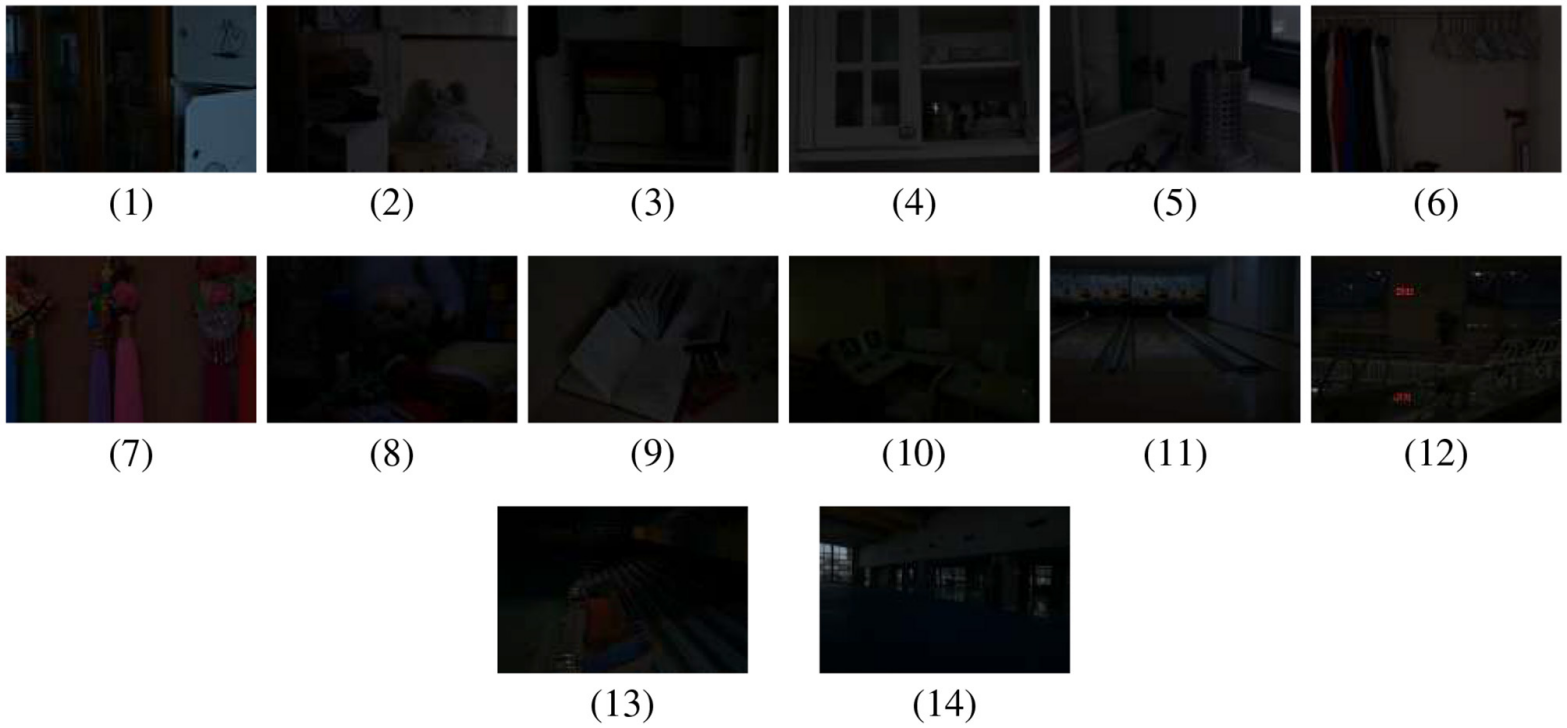
Original low-light images. Images reproduced from Chen et al. 2018 Deep Retinex Decomposition for Low-Light Enhancement dataset (https://daooshee.github.io/BMVC2018website/).

**Figure 3:**
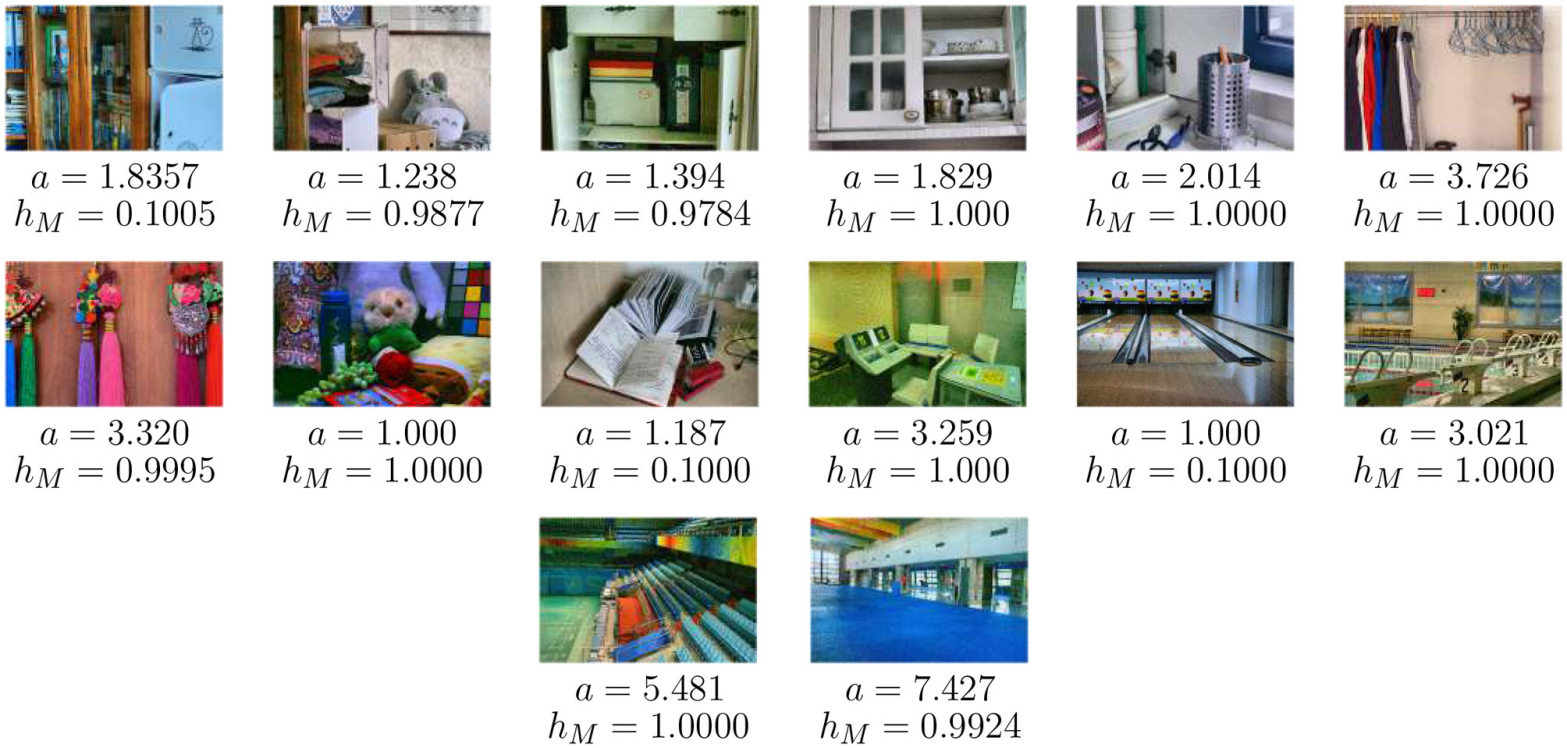
Enhanced images with optimized parameter values $\left(a^{*},h_{M}^{*}\right)$ obtained through RSA-based tuning. Images reproduced from Chen et al. 2018 Deep Retinex Decomposition for Low-Light Enhancement dataset (https://daooshee.github.io/BMVC2018website/).

The experiments were performed on a machine that have a 13 th-generation Intel^®^ Core™ i7-1360P processor (2.20 GHz), 16 GB RAM and a 475 GB SSD, running Windows 11 Home Single Language (version 24H2). All the implementations were done in MATLAB R2024b with Image Processing Toolbox. The assessment utilized LOL dataset (Wei et al., 2018), which contains 500 paired low-light and normal-light images. For this study, 14 low-light images were selected, as shown in Figure 2. The LOL dataset is a widely used benchmark for low-light image enhancement due to its acquisition noise and standardized 400 × 600 indoor scenes. The computational time (CT) of the proposed RSA-based framework, as shown in Figure 3 ranged between 120s and 150s with slight variations observed for the same image across different runs due to the stochastic nature of RSA. The dataset is publicly available for research purposes.

### 4.1 Parameter selection

The parameters that are used in the successful implementation of our proposed methodology are given in Table 1. The population size is set to be 100 candidate solutions, which represents a balanced approach between both computational efficiency and solution diversity. A lower risk number of population can cause premature convergence, excessively large population would be a very large source of computational overhead without proportional performance gains. The 100 iterations had been decided by the maximum. empirical analysis, which gave sufficient convergence on all test images at relatively low runtime reasonable. The exploration and exploitation parameters α = 0.1 and β = 0.9 were set to emphasize local refinement while maintaining sufficient global search capability, making this configuration particularly suitable for image enhancement applications requires a precise parameter tuning. The search space was limited to *a* ∈ [0.1, 10] and *h*_*M*_ ∈ [0.1, 1] depending on the theoretical characteristics of Interval-Valued Intuitionistic Fuzzy Sets and based on empirical observations from initial experiments (Jebadass and Balasubramaniam, 2024). These bounds guarantee the mathematical stability, but include the effective parameter set as various conditions of low-light enhancement.

**Table 1:** Parameter employed in RSA optimization.

**Parameter**	**Value**
Population size	100
Maximum iterations	100
α	0.1
β	0.9
*a* bounds	[0.1, 10]
*h*_*M*_ bounds	[0.1, 1]

### 4.2 Efficiency and precision advantages of RSA

The benefits of optimization that the proposed RSA framework offers are very evident in Table 2. Using As a representative example, Image 1, the RSA method was able to find the precise optimal parameter pair (*a* = 1.381, *h*_*M*_ = 0.1005), but the coarse and fine brute-force searches were essentially cannot take this step size solution because steps are discrete. Limited the rough brutality method to 0.1 change, only reached the suboptimal pair (*a* = 1.4, *h*_*M*_ = 0.1). Although the fine brute-force search provided a slightly closer to the truth, ′*a*′ parameter (*a* = 1.38), it was still a limited tool that was offered by brute-force search to *h*_*M*_ values (*h*_*M*_ = 0.10), which does not allow *h*_*M*_ to go to the real continuous optimum. This slight improvement needed a high cost of computation, which needed 100,000 evaluations and more than 11,600 s of processing time. As a comparison, RSA identified the exact optimal pair for Image 1 using only 3,000 evaluations in 130.4 s achieving 97% reduced appraisals and 98.9% lower calculation time. By working on a continuous parameter space, RSA achieves higher precision and improved efficiency. This performance advantage is consistently observed across all test images, as reflected in the varied optimal parameter combination $\left(a^{*},h_{M}^{*}\right)$ shown in Figure 3, confirming RSA's robustness and adaptability for multi-parameter, image-specific optimization.

**Table 2:** Comparative analysis demonstrating the optimization efficiency and parameter tuning precision of RSA.

**Method**	**Parameter ranges**	**Step / precision**	**Evaluations**	**Time (seconds)**	**Key finding**
Brute-force (Coarse) (Reference)	*a*: 1.0–10.0, *h*_*M*_: 0.1–1.0	0.1 (discrete)	910	98.5	Limited precision due to coarse grid; unable to reach exact optimum
Brute-force (Fine)	*a*: 1.0–10.0, *h*_*M*_: 0.1–1.0	0.01 (discrete)	100,000	11,658.6	High computational cost; still constrained by discrete steps
IVIFI-RSA (Proposed)	*a*: 1.0–10.0, *h*_*M*_: 0.1–1.0	Continuous	3,000	130.4	Efficient continuous optimization; precisely identifies optimal parameter (e.g., θ = 1.381)

### 4.3 Comparison of existing methods

The visual and performance analysis of the proposed enhancement method is demonstrated through its comparison with seven state-of-the-art methods: CLAHE (Haddadi et al., 2023), LIME (Supraja et al., 2022), LightenNet (Yang et al., 2023), FlightNet (Ozcan et al., 2023), AIE (Wang et al., 2019), SSIF (Demir and Kaplan, 2023), and IVIFI (Jebadass and Balasubramaniam, 2023). Here, CLAHE, LIME, LightenNet, FlightNet, AIE, SSIF and IVIFI denote the names of the enhancement methods, whereas the authors and years in brackets correspond to the original works.

Table 3 provides a comprehensive visual comparison across all 14 test images from the LOL dataset. The first column displays the original low-light images, followed by enhancement results from: CLAHE (second column), AIE (third column), FlightNet (fourth column), LightenNet (fifth column), LIME (sixth column), SSIF (seventh column), IVIFI (eighth column), and the proposed method (ninth column). This extensive comparison demonstrates the consistent performance of our approach across diverse lighting conditions and scene types.

**Table 3:** Visual comparison of various enhancement methods applied to all 14 test images in the LOL dataset.

**S.No**	**Original Image**	**CLAHE**	**LIME**	**LightenNet**	**FlightNet**	**AIE**	**SSIF**	**IVIFI**	**Proposed method**
1	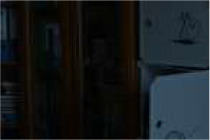	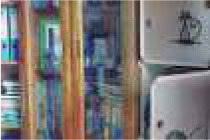	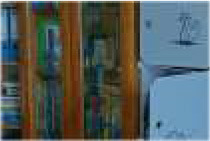	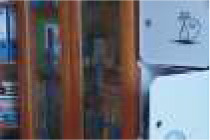	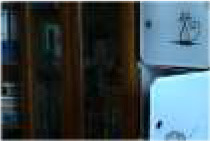	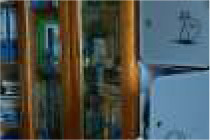	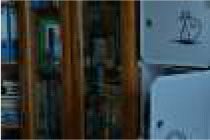	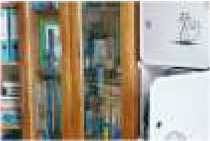	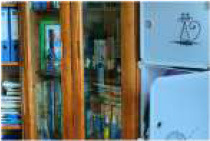
2	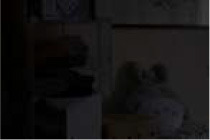	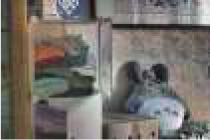	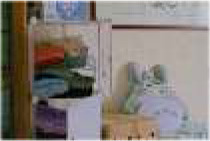	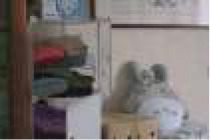	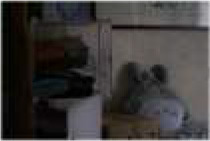	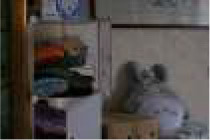	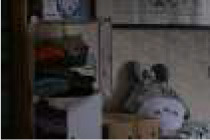	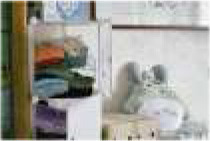	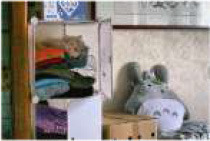
3	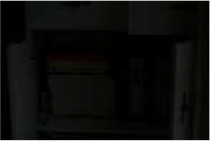	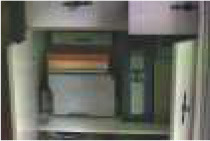	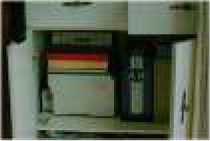	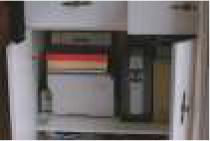	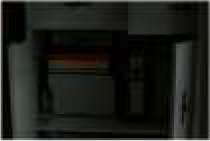	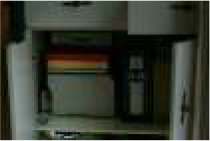	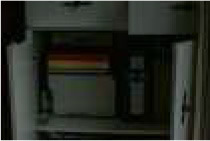	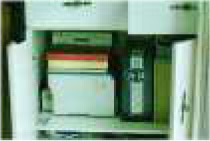	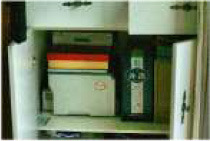
4	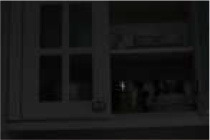	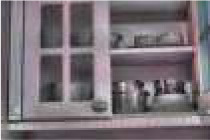	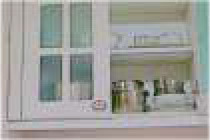	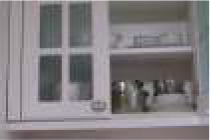	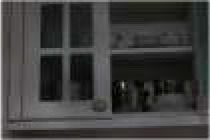	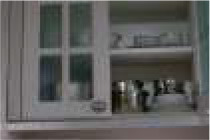	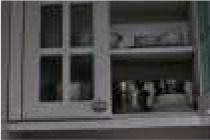	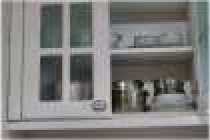	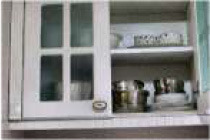
5	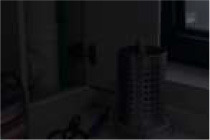	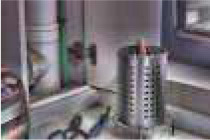	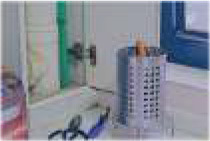	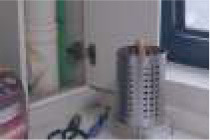	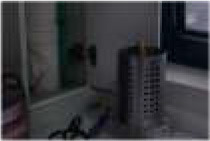	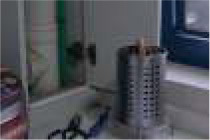	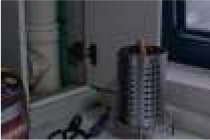	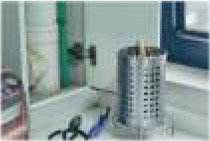	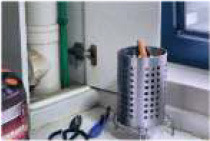
6	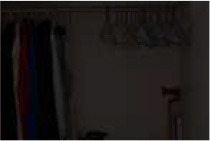	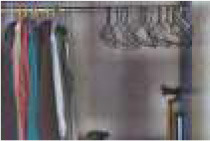	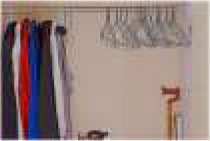	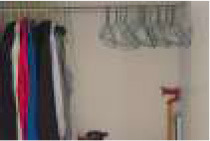	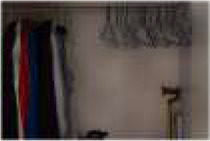	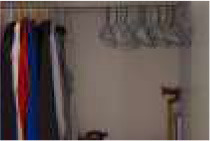	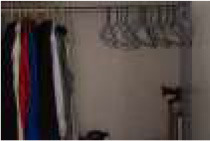	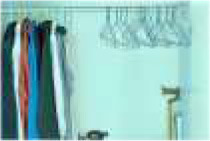	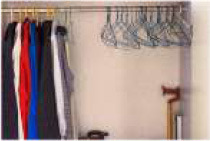
7	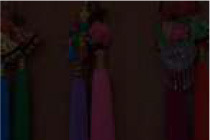	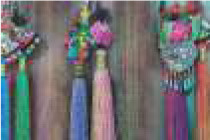	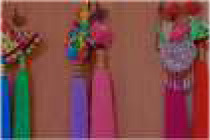	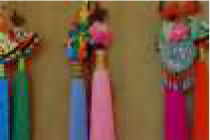	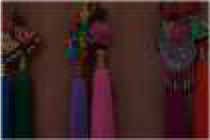	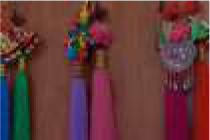	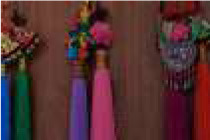	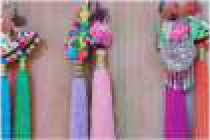	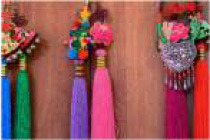
8	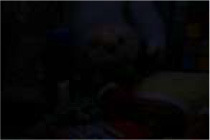	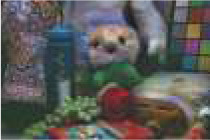	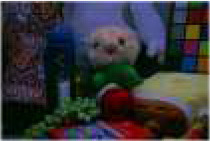	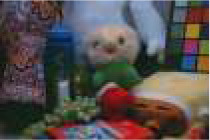	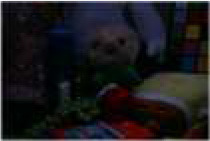	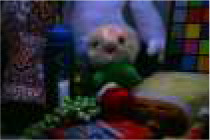	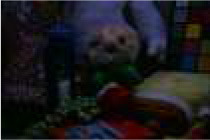	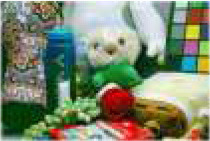	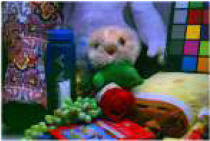
9	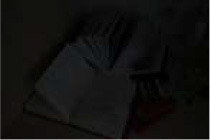	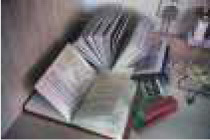	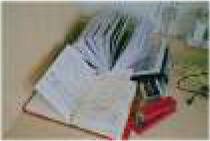	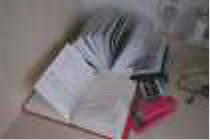	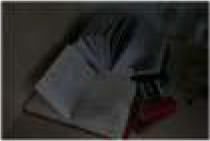	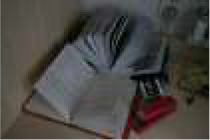	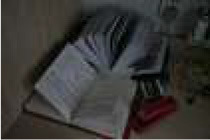	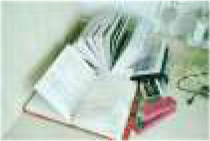	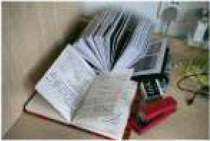
10	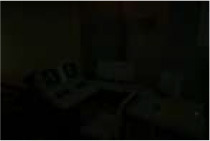	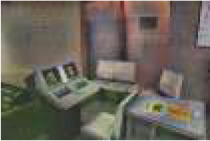	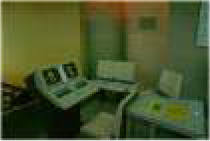	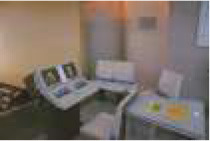	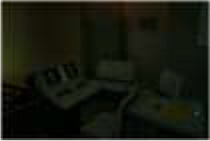	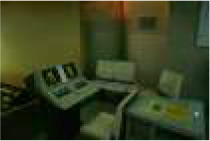	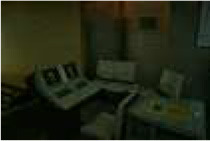	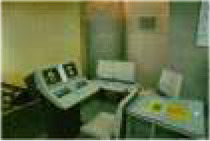	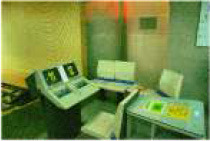
11	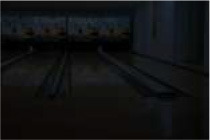	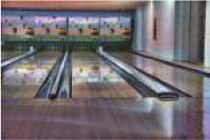	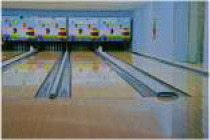	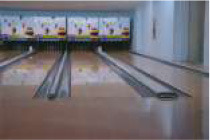	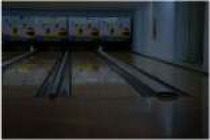	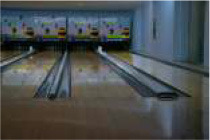	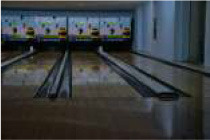	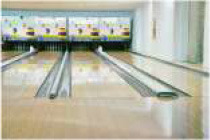	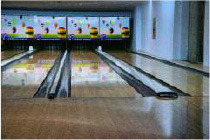
12	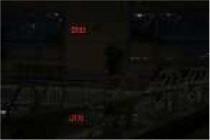	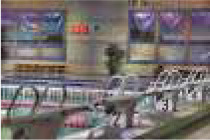	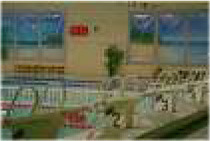	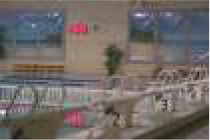	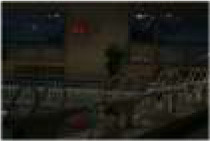	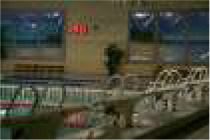	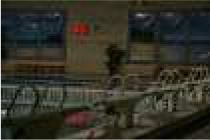	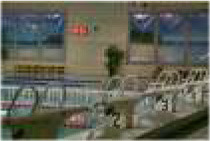	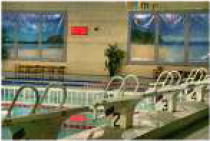
13	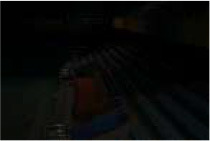	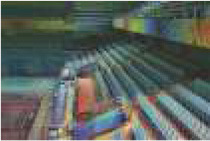	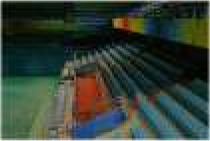	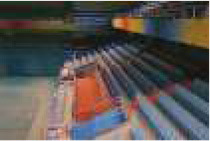	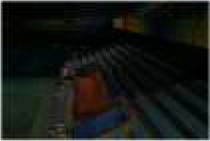	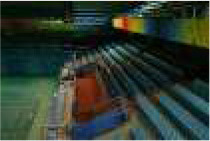	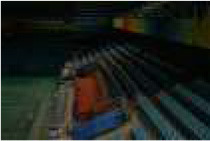	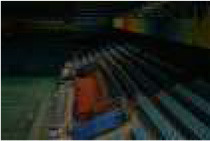	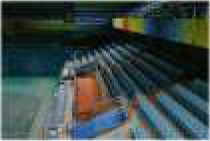
14	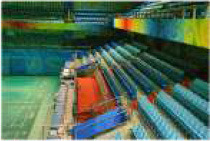	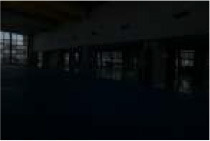	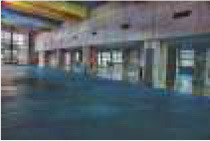	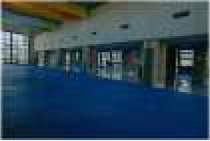	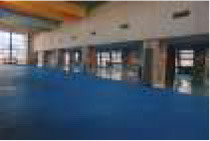	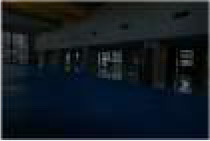	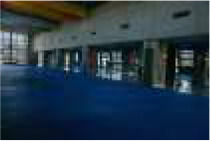	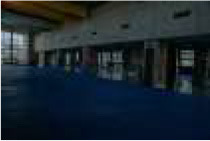	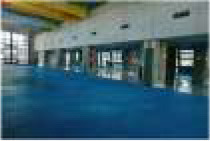

Images reproduced from Chen et al. 2018 Deep Retinex Decomposition for Low-Light Enhancement dataset (https://daooshee.github.io/BMVC2018website/).

### 4.4 Visual analysis

**Figure 4:**
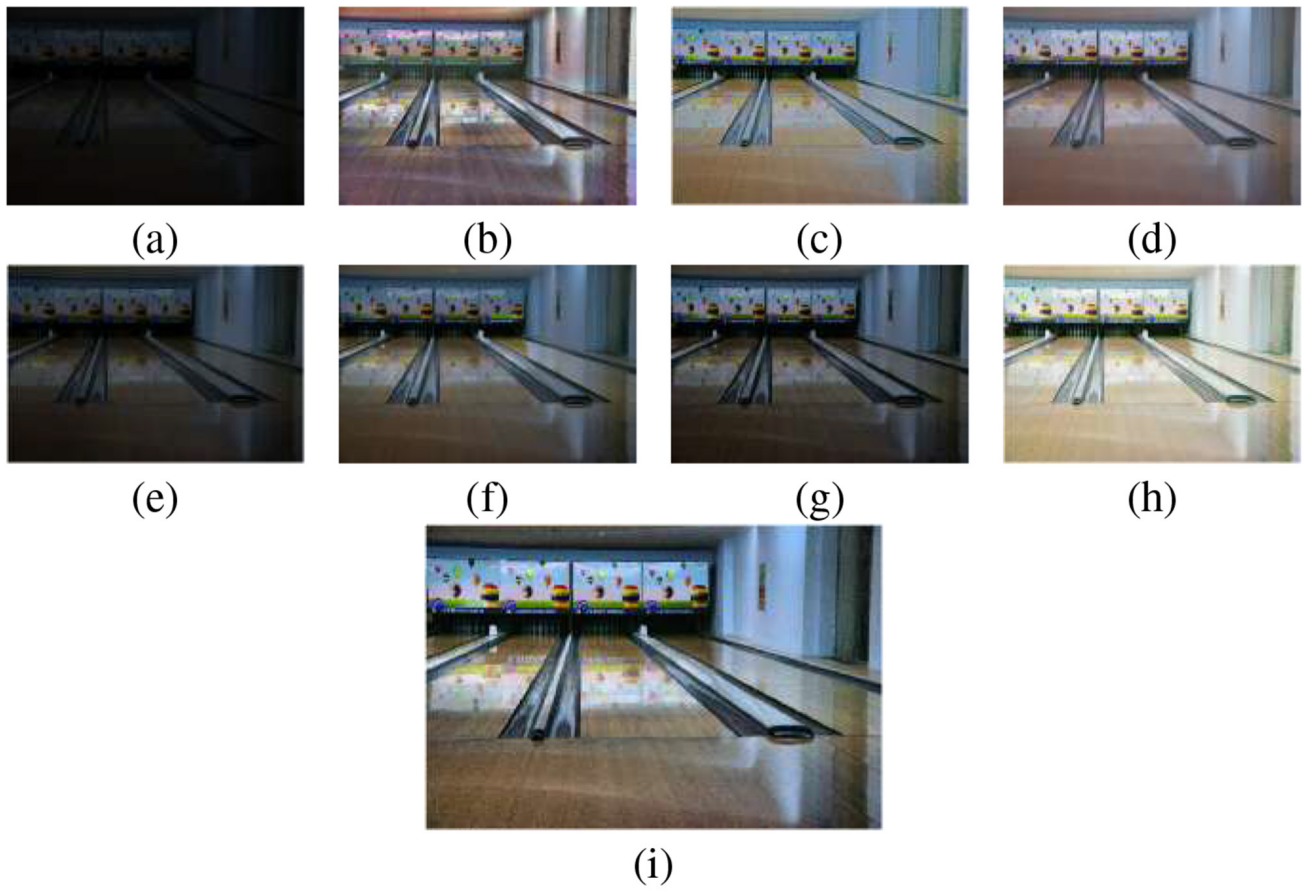
Visual comparison of various enhancement results for Image 11: **(a)** Original, **(b)** CLAHE, **(c)** AIE, **(d)** FlightNet, **(e)** LightenNet, **(f)** LIME, **(g)** SSIF, **(h)** IVIFI, and **(i)** Proposed. Images reproduced from Chen et al. 2018 Deep Retinex Decomposition for Low-Light Enhancement dataset (https://daooshee.github.io/BMVC2018website/).

**Figure 5:**
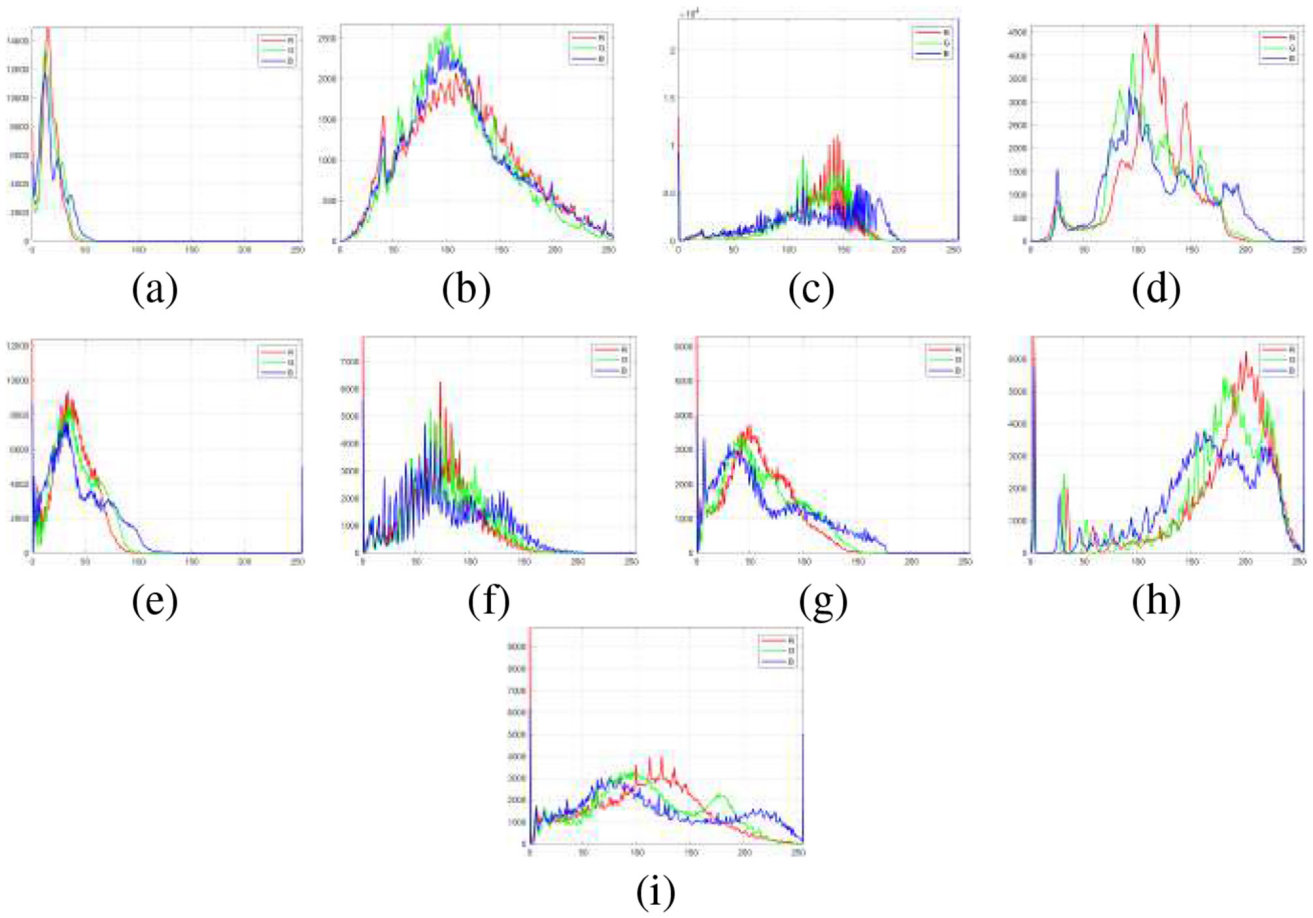
Histogram analysis of the Image 11 under various enhancement methods: **(a)** Original, **(b)** CLAHE, **(c)** AIE, **(d)** FlightNet, **(e)** LightenNet, **(f)** LIME, **(g)** SSIF, **(h)** IVIFI, and **(i)** Proposed.

In Figure 4, the enhancement results produced by various methods for Image 11, while Figure 5 shows the corresponding histogram equalization, in which the contrast distribution of the enhanced outputs is illustrated.

Figure 4 demonstrates the enhancement results of a low-light image: (a) the original image exhibits poor visibility with limited detail and dynamic range, (b) CLAHE improves local contrast and object visibility but introduces noise and reddish tones, (c) AIE brightens the image but causes overexposed areas and a bluish color cast, (d) FlightNet suppresses noise and smooths illumination but over-smooths textures, (e) Lightnet provides conservative enhancement, preserving natural tones while leaving shadows undercorrected, (f) LIME enhances details and contrast but produces halo artifacts and amplified noise, (g) SSIF improves structural clarity with natural exposure but slightly over-smooths and desaturates some regions, (h) IVIFI offers high contrast and vibrant colors with minor haloing and local noise and (i) the proposed method achieves balanced brightness, accurate colors, well-preserved textures and effective noise mitigation, demonstrating robustness for real-world low-light enhancement.

Figure 5 shows the histograms corresponding to Figure 4. (a) Original is compressed in the lower intensity range indicating poor brightness and contrast, (b) CLAHE depicts that pixel values are widely dispersed in the middle range of intensity, which means that the overall contrast could be increased. This behavior is also reflected in the corresponding image, where there is better brightness, as well as visible noise amplification in smooth areas, (c) AIE increases brightness and mid-to-high intensities but over-amplifies highlights, (d) FlightNet smooths midtones and preserves details but under-enhances shadows, (e) Lightnet enhances dark regions while offering limited contrast in bright areas, (f) LIME improves dark and midtones with noise control but struggles in severely underexposed regions, (g) SSIF enhances textures but may introduce noise, (h) IVIFI balances midtones and highlights with good color preservation, though fine details may blur and (i) Proposed method produces a well-distributed histogram across all channels. The red channel shows distinct peaks in the low-to-mid range, enhancing contrast and details, while green and blue channels remain uniform, preserving color fidelity. This balanced distribution demonstrates effective contrast enhancement without over-saturation or loss of detail.

### 4.5 Performance analysis

Four complementary measures are taken to make sure that enhancement performance is assessed thoroughly used: Entropy to maintain detail preservation (Ma et al., 2022), CII to imprive contrast (Mittal et al., 2019), AMBE for brightness consistency (Ozturk and Ozturk, 2023), a PSNR for noise reduction and fidelity (Veluchamy and Subramani, 2023) and a NIQE (Natural Image Quality Evaluator) uses measurable changes from statistical regularities of natural images to measure image quality, and small values indicate better performance (Du et al., 2022). The average values of all of these metrics are displayed in Table 4. A balanced analysis is ensured through this multi-factor evaluation, exceeding what any individual metric can provide. For each metric, the percentage improvement of the proposed method over a baseline is calculated using the standard formula:


$$\begin{array}{c} \text{Improvement (\%)}=\left(\frac{M_{\text{prop}}-M_{\text{base}}}{\left|M_{\text{base}}\right|}\right)\times 100 \end{array}$$ (41)

Here, *M*_prop_ and *M*_base_ denote the metric values corresponding to the proposed and baseline methods, respectively. In all result tables, the best and second-best performances are used to indicate in blue and red, respectively.

**Table 4:** Average quantitative metrics for different image enhancement methods.

**Methods / metrics**	**CLAHE**	**LIME**	**LightenNet**	**AIE**	**FlightNet**	**SSIF**	**IVIFI**	**Proposed**
Entropy	6.8067	6.8603	6.2122	6.9880	6.8272	6.6248	7.3297	**7.6000**
AMBE	91.2611	52.4487	26.8022	92.8022	90.1863	35.1063	102.3933	**124.6198**
CII	4.7541	4.7985	2.7379	0.3820	7.7856	3.3868	6.2965	**7.4611**
PSNR	8.2215	12.7574	16.3185	8.5380	7.8102	16.0870	13.2478	**20.1735**
NIQE	9.3693	8.4769	6.0428	7.9032	7.9129	8.6038	7.3073	**3.7678**

Bold values indicate the best performance among the compared methods.

#### 4.5.1 Entropy analysis

Entropy is taken as an important measure of the degree of information and detail retained in an enhanced image, which increases values signify increased texture preservation and better perceptual quality. The Shannon entropy formulation that is used in the present piece of work is (25). The entire set of entropy calculation in Table 5 provides the values for each test image across all compared methods, whereas the following discussion focuses on the average performance derived from this comprehensive data. The Analysis of the average values of entropy show there is a distinct performance pyramid. Traditional methods such as CLAHE (6.81) and LIME (6.86) shows a moderate improvement compared to the original images which have a average of 4.95. Among deep learning mehtods, LightenNet (6.21) and SSIF (6.62) have more conservative gains, while FlightNet (6.99) and AIE (6.83) are more effectively used when it comes to information. The IVIFI method attains a average entropy of 7.33, which significant improvement. The proposed RSA-optimized method has the largest average entropy value of 7.63, which shows that it preserves better processing and improves information in dim surroundings. This increase is by 53.5% over the original image and a 3.69% improvement compared to the IVIFI method, which underlines its effectiveness in maximizing visual information to a variety of lighting conditions.

**Table 5:** Entropy-based performance analysis.

**S.NO**	**Original image**	**CLAHE**	**LIME**	**LightenNet**	**FlightNet**	**AIE**	**SSIF**	**IVIFI**	**Proposed method**
1	5.8571	7.4826	7.1568	6.8774	7.0128	6.8800	6.9857	7.6156	7.8648
2	5.2868	7.2051	6.9996	6.8141	7.1414	7.0783	6.9460	7.4917	7.7308
3	4.5626	7.4364	6.9094	6.0395	7.3309	7.2654	6.4087	7.7746	7.4741
4	5.2337	7.4660	6.9074	6.7699	6.9199	6.5487	7.1016	7.2517	7.7224
5	5.2252	7.4214	6.9362	6.6369	7.0156	6.7898	6.9025	7.3067	7.7290
6	5.1772	7.2401	6.6963	6.7933	6.6951	6.4769	6.9178	6.8940	7.6871
7	5.4264	7.2514	6.4008	5.9656	6.6198	6.3513	6.6226	7.1004	7.7763
8	4.1892	7.0069	6.7545	5.8556	7.1480	6.8594	5.9390	7.8607	7.1379
9	4.9988	7.5208	6.7611	6.2624	7.0050	6.9508	6.7182	7.0730	7.6833
10	4.2579	7.1431	6.7473	5.4547	6.8079	6.8084	6.1077	7.2692	7.5404
11	5.1977	7.4261	6.9716	6.3283	7.0368	6.8128	6.9609	7.1683	7.7598
12	4.7943	7.4543	7.0333	5.8903	6.9026	6.9649	6.6487	7.2164	7.6815
13	4.3397	7.1795	6.9757	5.6494	7.2324	7.0095	6.1626	7.2862	7.1876
14	4.7869	7.1056	6.7943	5.6336	6.9641	6.7847	6.3255	7.3067	7.4250

Blue indicates the best value and red indicates the second-best value among the compared methods.

#### 4.5.2 Absolute Mean Brightness Error (AMBE) analysis

The Average Mean Brightness Error (AMBE) metric is used to measure of the difference in brightness between original and enhanced images, and the value of which is computed in (28). The entire set of AMBE calculation in Table 6 provides the values for each test image across all compared methods, whereas the following discussion focuses on the average performance derived from this comprehensive data. Lower AMBE values indicate better preservation of original brightness levels, whereas higher values reflect more significant enhancement aimed at improving visibility. Analysis of the average AMBE scores represents that LIME (52.45) offers moderate performance in terms of improving. Deep learning methods are varying in their effectiveness: LightenNet (26.80) and SSIF (35.08) show conservative brightness adjustment, while CLAHE (91.26), FlightNet (90.19) and AIE (92.04) achieve more pronounced transformation. The IVIFI method produces a significant brightness shift with an average AMBE of 102.39. The maximum average AMBE is 124.62 which is registered by the optimized form of RSA approach, which indicates that regular and high-quality brightness enhancement is acquired in varying low light situations. This is equivalent to an increase of 204.62% over the high capability of its own and a 21.71% improvement relative to the IVIFI procedure, it is good at original images to increase brightness conversion and keep visual balance and retain a significant image structures.

**Table 6:** AMBE-based performance analysis.

**Original images**	**CLAHE**	**LIME**	**LightenNet**	**AIE**	**FlightNet**	**SSIF**	**IVIFI**	**Proposed method**
Image 1	94.1688	53.927	46.187	62.861	81.54	37.515	94.1335	104.104
Image 2	90.0018	55.612	37.295	110.78	97.139	40.301	60.4309	132.6008
Image 3	95.7955	43.531	17.665	84.046	95.243	28.022	73.9842	132.9822
Image 4	92.7573	70.183	38.997	138.77	104.05	50.493	118.8621	126.1099
Image 5	92.4349	59.89	34.357	123.06	96.802	45.288	71.6359	127.603
Image 6	82.7436	59.327	47.125	114.5	95.955	46.247	127.152	137.2734
Image 7	90.9278	48.788	23.572	77.485	73.701	37.342	81.206	102.5372
Image 8	83.8941	39.777	17.398	54.434	74.011	19.467	104.451	129.2907
Image 9	100.5230	54.451	26.178	122.6	98.251	38.723	18.7991	134.6397
Image 10	90.3898	48.374	14.366	74.859	104.73	24.555	177.907	137.3341
Image 11	93.6824	58.988	25.923	110.14	95.15	42.228	41.5167	114.0094
Image 12	100.2981	57.439	17.823	94.984	94.067	34.944	171.917	124.4918
Image 13	86.2103	43.38	13.911	58.644	75.301	21.656	163.279	108.3907
Image 14	83.8282	40.617	14.435	61.341	76.668	24.298	128.2314	133.3104

Blue indicates the best value and red indicates the second-best value among the compared methods.

#### 4.5.3 Contrast Improvement Index (CII) analysis

The Contrast Improvement Index (CII) was reported the effectiveness of an enhancement methods in improving image contrast by characterizing the ratio between the enhanced image and original image mean intensities. A Value of CII above 1 is indicative of successful contrast improvement and values less than 1 are not indicative of success to express contrast degradation and the formula which involves calculation of the CII is defined in (26). Table 7 presents reported CII values of all the test image across all methods, and the following analysis focuses on the average performance across the dataset. The average CII values illustrate that traditional methods including CLAHE (4.75) and LIME (4.80) provide noticeable contrast improvement. Varied performance is are found in the deep learning frameworks like LightenNet (2.74) and AIE (0.39) shows weak, whereas FlightNet (7.79) has significant improvement. The IVIFI method attains an average CII of 6.30, confirming the utility of intuitionistic fuzzy approaches. The proposed RSA-optimized method is having better performance with average CII of 7.48. In direct comparison with the original images and IVIFI method 18.73% improvement compared to the IVIFI method, and 85.35% attained by the IVIFI method surpassing the original pictures, making it obvious that it is good.

**Table 7:** CII-based performance analysis.

**Original images**	**CLAHE**	**LIME**	**LightenNet**	**AIE**	**FlightNet**	**SSIF**	**IVIFI**	**Proposed method**
Image 1	1.9000	3.1886	2.8745	0.4492	4.3093	2.5226	3.5421	3.0225
Image 2	5.5225	3.8719	2.926	0.2957	6.0165	3.0812	6.1098	5.6891
Image 3	5.9366	5.4695	2.8137	0.3759	10.779	3.8771	11.0626	9.665
Image 4	4.3806	4.1554	2.7533	0.2269	5.6782	3.2701	3.6629	7.1458
Image 5	3.9687	4.102	2.7795	0.3097	6.0138	3.3457	3.703	6.5315
Image 6	2.9111	3.4353	2.9344	0.2642	4.9387	2.8984	4.8222	5.8951
Image 7	5.0761	3.4514	2.1844	0.4688	4.7031	2.8762	6.1811	7.648
Image 8	5.7972	7.2017	3.7125	0.5102	12.539	4.0351	14.2809	10.0432
Image 9	5.8363	4.443	2.6553	0.274	7.2125	3.4484	7.2121	7.9589
Image 10	7.268	6.5655	2.6528	0.4464	13.049	3.8251	7.7912	13.3013
Image 11	5.0430	4.4372	2.5106	0.334	6.5444	3.4606	6.1284	7.221
Image 12	4.8008	5.3421	2.3473	0.34	8.1111	3.6416	3.6458	6.5404
Image 13	5.4137	6.6519	2.8125	0.518	10.811	3.8215	6.8146	8.7656
Image 14	2.7035	4.8637	2.3731	0.5354	8.293	3.3114	3.1937	5.0274

Blue indicates the best value and red indicates the second-best value among the compared methods.

#### 4.5.4 Peak Signal-to-Noise Ratio (PSNR) analysis

The PSNR is a widely known measure of the noise level regarding an improved image. Higher PSNR values show improved noise elimination and improved image quality. It is defined as


$$\begin{array}{c} PSNR=20\log_{10}\left(\frac{L}{\sqrt{\frac{1}{MN}\sum_{i=0}^{M-1}\sum_{j=0}^{N-1}{\left[O\left(i,j\right)-E\left(i,j\right)\right]}^{2}}}\right) \end{array}$$ (42)

where *L* represents the maximum possible pixel value, *M* and *N* are the image dimensions, *O*(*i, j*) denotes the pixel value at position (*i, j*) in the original image and *E*(*i, j*) is the corresponding pixel in the enhanced image. Table 8 presents the complete set of PSNR results calculated for each individual test image across all compared methods. The discussion below focuses on the average PSNR values derived from this comprehensive dataset, which offer a more robust evaluation of each method's overall performance than single-image results. The average PSNR values indicates clear performance across method categories. Traditional methods including CLAHE (8.22) and LIME (12.76), demonstrate only modest quality improvements. Among deep learning methods, performance shows noticeable improvement: LightenNet (16.32) and SSIF (16.17) achieve acceptable noise reduction, while AIE (8.53) and FlightNet (7.81) performs poorly for low-light enhancement. The IVIFI method obtains a moderate PSNR of 13.24, indicating better performance than the weaker deep learning approaches. The proposed RSA-optimized method yields the highest average PSNR of 20.11, indicating its superior noise reduction ability. When compared directly with the original images and the IVIFI method, it achieves a 28.96% improvement over the original images and 66.12% enhancement over the IVIFI method, demonstrating the strong capability of the proposed approach to enhance image quality while minimizing noise amplification in low-light conditions.

**Table 8:** PSNR-based performance analysis.

**Original images**	**CLAHE**	**LIME**	**LightenNet**	**AIE**	**FlightNet**	**SSIF**	**IVIFI**	**Proposed method**
Image 1	7.8948	12.613	11.305	9.0256	9.9016	15.35	12.2252	17.8832
Image 2	8.3909	12.298	14.214	7.7281	5.5144	14.488	8.7259	12.8039
Image 3	7.8573	13.482	17.755	7.7729	7.3872	17.174	9.369	17.8061
Image 4	7.9227	10.805	14.382	7.5112	4.2575	12.898	17.0933	20.1921
Image 5	8.0091	11.924	15.303	8.0683	5.957	14.024	10.8212	20.3782
Image 6	9.0605	11.957	12.966	7.9454	4.6454	13.561	5.7544	18.407
Image 7	8.2806	13.875	17.427	10.407	10.0064	15.691	11.5146	27.8109
Image 8	9.0229	13.843	17.784	9.728	11.0764	19.895	10.9425	19.3744
Image 9	7.3029	12.791	16.842	7.9682	5.1641	15.308	5.4528	19.4576
Image 10	8.4473	13.392	18.693	7.3978	9.6268	19.044	24.6462	18.9882
Image 11	7.8820	12.041	16.843	8.2298	6.6445	14.361	6.1284	23.1161
Image 12	7.3723	12.102	18.081	8.407	6.7478	16.022	24.5849	21.309
Image 13	8.7084	13.167	18.468	9.5778	10.9155	18.863	22.1549	24.1714
Image 14	8.9498	14.313	18.396	9.7646	11.4989	18.539	16.0562	20.7313

Blue indicates the best value and red indicates the second-best value among the compared methods.

## 5 Limitations and applications

The proposed methodology has a few number limitations that must be noted:

- The algorithm has a relatively intense computational cost involved compared with the conventional enhancement techniques, which might be not applicable in real-time on limited processing capability devices can be used.
- This study has evaluated the proposed method only on static images, which represents a major limitation. Although it is theoretically feasible to modify the RSA framework to improve the video enhancement, experimentation has not verified this capability. As a result, any mentioned video applications should be noted as theoretical rather than verifiable, and future work will focus on incorporating temporal information and validating this approach on video datasets.
- Performance is based on parameter ranges which are configured and might require minor change to fit some imaging conditions.
- Since this method is offered particularly to optimize the image enhancement of the low-light and could not be easily generalized to other image processing domains.

The proposed method demonstrates strong potential for various practical applications:

- Medical imaging: Medical image improvement in low-light images and endoscopic image.
- Surveillance systems: Enhancing clarity of detail in night security surveillance.
- Autonomous navigation: Consistent perception of the surroundings in low light.
- Astronomical imaging: An in-depth examination of the heavenly bodies.

## 6 Conclusion and future direction

This paper introduced an efficient Interval-Valued Intuitionistic Fuzzy Image (IVIFI) was introduced to optimized the enhancement framework by using the Reptile Search Algorithm (RSA). By adaptively selecting parameters of fuzzy membership and fuzzy hesitation, the suggested approach increases brightness, contrast and fineness low-light image structures and maintaining natural visual image quality. Quantitative evaluations based on the use of Entropy, AMBE, PSNR and CII indicate that the proposed method is always better as compared to conventional techniques, generating both visually good and analytically sound results. Owing to its adaptability, the architecture is suited to a wide variety of real-world tasks including surveillance, medical imaging and remote sensing in which strong image improvement under adverse lighting situations is necessary.

This method can be generalized in the future,

- While the suggested RSA-driven IVIF method performs well on static images, its applicability to video enhancement had not been evaluated within the scope of this study. This approach can be extended to video sequences using either frame-wise optimization or temporal coherence, which is still a promising avenue for future research. Our future research will include an analysis of such extensions.
- The optimization process can be further enhanced by exploring alternative metaheuristic algorithms or hybrid metaheuristic approaches to computational efficiency, enhance global convergence and overall performance.
- Experiments on large-scale, real-world datasets of a variety of illumination environmental conditions high lights the strength of the suggested method.

In conclusion, the RSA–IVIF framework marks a significant advancement in adaptive low-light image enhancement which provides consistent high-quality outcomes.

## Data Availability

Publicly available datasets were analyzed in this study. This data can be found here: https://daooshee.github.io/BMVC2018website/.
